# *rs41291957* controls miR‐143 and miR‐145 expression and impacts coronary artery disease risk

**DOI:** 10.15252/emmm.202114060

**Published:** 2021-09-22

**Authors:** Ignacio Fernando Hall, Montserrat Climent, Chiara Viviani Anselmi, Laura Papa, Vinicius Tragante, Luca Lambroia, Floriana Maria Farina, Marcus E Kleber, Winfried März, Carlo Biguori, Gianluigi Condorelli, Leonardo Elia

**Affiliations:** ^1^ Humanitas Research Hospital‐IRCCS Rozzano Italy; ^2^ Institute of Genetics and Biomedical Research National Research Council Rozzano Italy; ^3^ Department of Cardiology Division Heart and Lungs University Medical Center Utrecht Utrecht University Utrecht The Netherlands; ^4^ Department of Molecular and Translational Medicine University of Brescia Brescia Italy; ^5^ Institute for Cardiovascular Prevention (IPEK) Ludwig‐Maximillians‐Universität (LMU) München Munich Germany; ^6^ Department of Medical Biotechnology and Translational Medicine University of Milan Milan Italy; ^7^ V^th^ Department of Medicine Medical Faculty Mannheim Heidelberg University Mannheim Germany; ^8^ SYNLAB Academy SYNLAB Holding Deutschland GmbH Augsburg and Mannheim Germany; ^9^ Clinical Institute of Medical and Chemical Laboratory Diagnostics Medical University Graz Graz Austria; ^10^ Interventional Cardiology Unit Mediterranea Cardiocentro Naples Italy; ^11^ Department of Biomedical Sciences Humanitas University Pieve Emanuele Italy

**Keywords:** atherosclerosis, coronary artery disease, genetics, microRNA, SNP, Biomarkers, Cardiovascular System

## Abstract

The role of single nucleotide polymorphisms (SNPs) in the etiopathogenesis of cardiovascular diseases is well known. The effect of SNPs on disease predisposition has been established not only for protein coding genes but also for genes encoding microRNAs (miRNAs). The miR‐143/145 cluster is smooth muscle cell‐specific and implicated in the pathogenesis of atherosclerosis. Whether SNPs within the genomic sequence of the miR‐143/145 cluster are involved in cardiovascular disease development is not known. We thus searched annotated sequence databases for possible SNPs associated with miR‐143/145. We identified one SNP, *rs41291957* (G > A), located −91 bp from the mature miR‐143 sequence, as the nearest genetic variation to this miRNA cluster, with a minor allele frequency > 10%. *In silico* and *in vitro* approaches determined that *rs41291957* (A) upregulates miR‐143 and miR‐145, modulating phenotypic switching of vascular smooth cells towards a differentiated/contractile phenotype. Finally, we analysed association between *rs41291957* and CAD in two cohorts of patients, finding that the SNP was a protective factor. In conclusion, our study links a genetic variation to a pathological outcome through involvement of miRNAs.

## Graphical abstract

The role of single nucleotide polymorphisms (SNPs) in the etiopathogenesis of cardiovascular diseases is well known. Here, we link a genetic variation to a pathological outcome through the study of pivot miRNAs involved in atherosclerosis development.

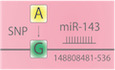

**The paper explained**

**Problem**

The rates of Coronary artery disease (CAD), defined as atherosclerosis of the coronary arteries and its lethal comorbidities are the most common cause of mortality worldwide. A hallmark of atherosclerosis is neointimal formation, that depends to the hyperplasia of vascular smooth muscle cells (VSMCs), which induces a gradual narrowing of vessels that could lead to vessel occlusion. It is common knowledge, that VSMCs can undergo a plethora of phenotypical transformations modulating their phenotype from contractile/differentiate to proliferative/dedifferentiated in response to pathological stimuli. Despite this connection, the role of genetics in VSCM phenotypic transition and CAD development in very limited.

**Results**

An *in silico* analysis led us to select a genetic variation (*rs41291957*) located in the miR‐143/145 locus, which encodes a microRNA cluster known to be a fundamental player in VSMC phenotypic switch, leading to atherosclerosis development. Through bioinformatics, ectopic and genetic approaches, such as CRISPR/Cas9, and analysis of primary VSMCs from healthy donors, we demonstrated that *rs41291957* positively modulates the expression of mature miR‐143 and miR‐145, altering the secondary structure of the related primary miRNA, thus increasing VSMC contractile/differentiation status. Then, we looked for possible association of *rs41291957* in two different and independent populations of patients with stable coronary artery disease (CAD) and demonstrated how expression of the variant is associated with protection against chronic total occlusion and CAD.

**Impact**

Besides the evaluation of clinical variables, therapeutic decisions in CAD should also take into account genetic characteristics of the patient. The *rs41291957* A‐allele variant could be a useful marker for CAD prognosis, therefore possibly represents a prognostic factor, helping the management of this disease.

## Introduction

Coronary artery disease (CAD), or atherosclerosis of the coronary arteries, is the most common cause of mortality worldwide (Roger, 2007). The pathogenesis of CAD is complex and involves the interaction between circulating inflammatory cells (i.e., monocyte‐derived macrophages) and resident vascular cells (i.e., endothelial cells [ECs] and vascular smooth muscle cells [VSMCs]) (Libby, 2002; Weber & Noels, 2011), eventually resulting in plaque formation and coronary occlusion. CAD manifests with a wide spectrum of anatomical phenotypes, ranging from isolated, single coronary lesions to diffuse, multivessel disease with chronic total occlusion (CTO) (Glass & Witztum, 2001; Stone *et al*, 2005).

MicroRNAs (miRNAs, miRs) are short, non‐coding RNAs that regulate gene expression through either cleavage of mRNA targets or inhibition of protein translation (Bartel, 2004). Their role in cardiovascular (CV) diseases, including atherosclerosis (Quintavalle *et al*, 2011; Elia & Condorelli, 2015), CAD (Fichtlscherer *et al*, 2010; Elia & Quintavalle, 2017), acute myocardial infarction (MI) (Wang *et al*, 2010), heart failure (Goren *et al*, 2012) and hypertrophic cardiomyopathy (Roncarati *et al*, 2014), has been widely described. Among the different miRNAs involved in vascular pathologies (Stratton *et al*, 2019), the bicistronic miR‐143/145 cluster plays a major role in vessel development and vascular diseases, including atherosclerosis and pulmonary hypertension, through a proliferative effect on VSMCs and ECs (Boettger *et al*, 2009; Cordes *et al*, 2009; Elia *et al*, 2009, 2018; Xin *et al*, 2009; Quintavalle *et al*, 2010; Climent *et al*, 2015). As a consequence, relatively small variations in miR‐143 and miR‐145 levels can dramatically affect vascular homeostasis and CAD development (Faccini *et al*, 2017).

Recent studies have identified single nucleotide polymorphisms (SNPs) on miRNA‐containing loci; known as miR‐SNPs, they can modify either the processing of primary/precursor‐miRNAs or the binding of mature miRNAs to mRNA targets in an allelic‐specific fashion (Saunders *et al*, 2007; Mishra & Bertino, 2009). Different SNPs have been studied for their potential roles in the regulation of miRNAs and then cardiovascular diseases, and this list includes miR‐126, miR‐146, and miR‐155, among others (Elfaki *et al*, 2019). However, knowledge on the role of genetic variations in miR‐143/145 biology is limited, so it is conceivable that a miR‐SNP exists that influences the cluster’s expression and, consequently, CAD development. Here, we describe the biological properties, including the prognostic value in human CV diseases, of a miR‐143/145 SNP that we identified as significant, namely, *rs41291957*.

## Results

### Identification of SNP *rs41291957* in the miR‐143/145 locus

**Figure 1:**
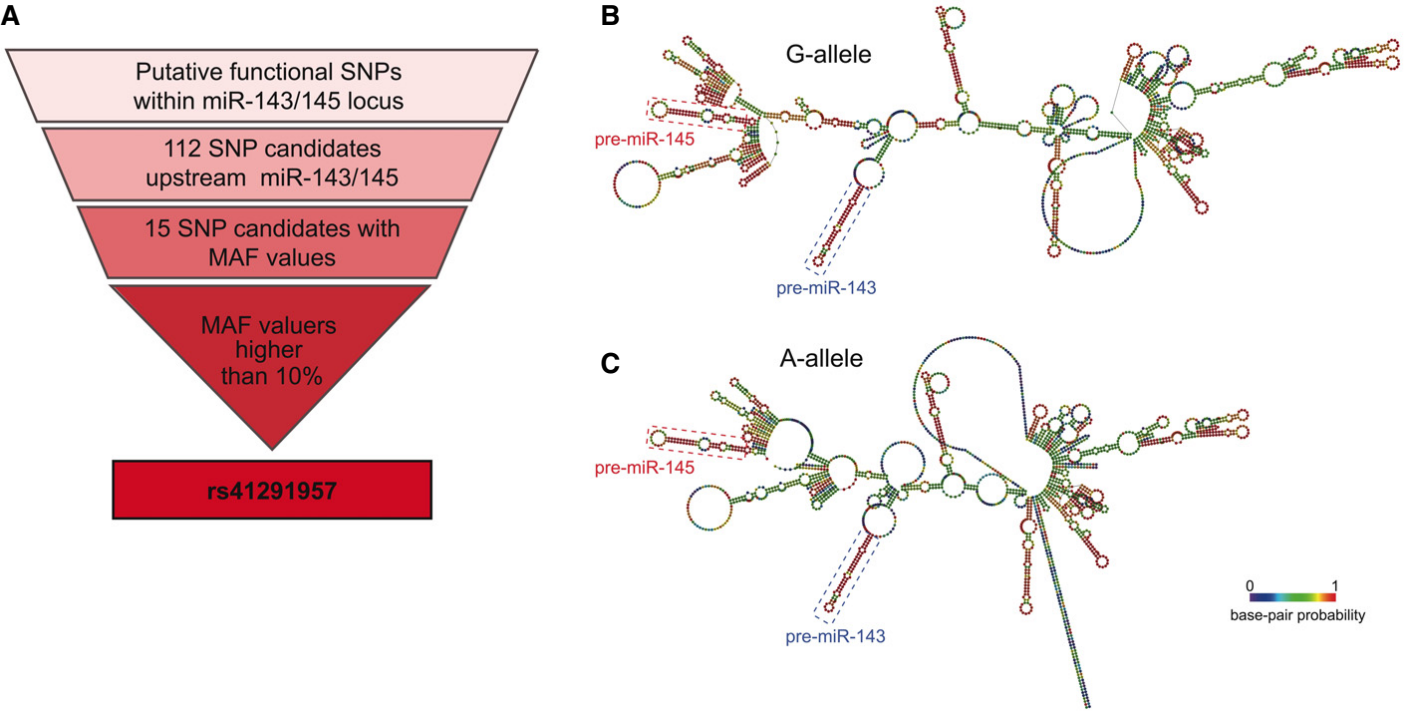
*rs41291957* selection AGraphic representation of the workflow used to select rs41291957.B, CSecondary structure prediction of the primary‐miR‐143/145 sequence, based on structural models of centromeric predictions of the miRNA primary sequences carrying the G‐ or A‐allele, based on base pair annealing probability (coloured boxes identify the precursor sequences for miR‐143 and miR‐145).

To uncover possible SNPs at the miR‐143/145 locus, we interrogated a public sequence database (ensembl.org), searching for variations located in the locus of interest. We focused on the first 300 bp upstream of miR‐143, the first gene of the cluster, identifying 112 SNPs, of which only 15 presented available minor allele frequency (MAF) values. We then filtered for variations with a MAF higher than 10%, identifying only one SNP with such a characteristic and, so, deemed of interest for further study: *rs41291957* (G > A; distance: −91 from the mature miR‐143 sequence) (Fig 1A and Dataset EV1).

### Secondary structure prediction

**Figure EV1:**
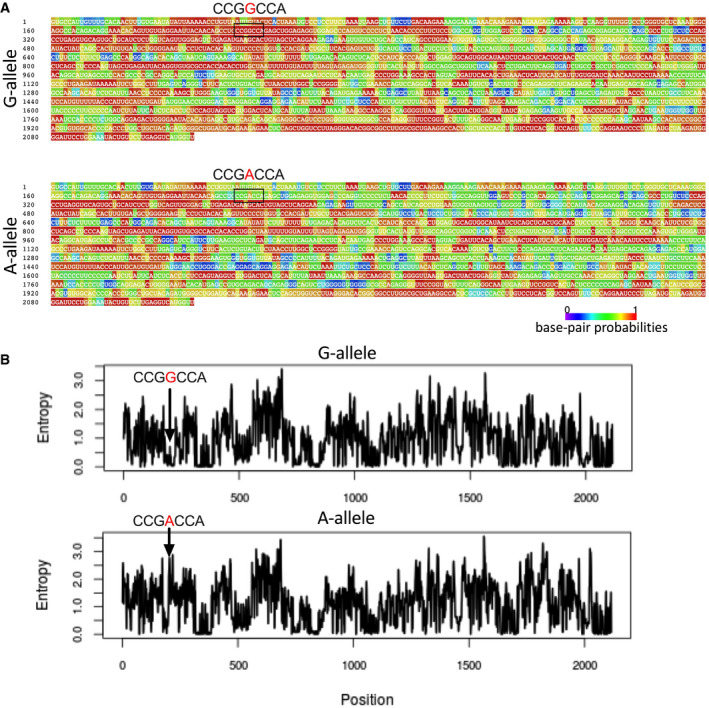
Thermodynamic analysis of the miR‐143/145 primary sequence ABase pair annealing probability using a centromeric prediction algorithm was carried out for the G‐ and A‐allele sequences (the base in red indicates the variation).BEntropy level profiles of the G‐ and A‐allele transcripts.

The miR‐143/145 cluster’s promoter is located −3 to −4 kb from the primary miRNA sequence (Cordes *et al*, 2009; Xin *et al*, 2009), whereas *rs41291957* is localized within the miR‐143/145 primary transcript. So, it is conceivable that this variation has no influence on transcription of the miRNA cluster. We therefore asked whether *rs41291957* might modulate the structure of this long transcript. With this aim, we performed *in silico* prediction of the cluster’s secondary structures for the reference (G) and minor (A) alleles. A significant difference in minimum free energy between the primary miRNAs of the two alleles was found (ΔG: G‐allele, −594.32 kcal/mol; A‐allele, −499.86 kcal/mol). The lower minimum free energy of the A‐allele highlighted a potentially less complex structure with greater accessibility for miRNA maturation enzymes (Fig 1B). Accordingly, the base pair probability values of the A‐allele were, in general, lower than those of the G‐allele (Fig EV1A). Moreover, the A‐allele had increased potential molecular entropy (Fig EV1B), a finding that could explain the altered loop formation at the beginning and end of the primary miRNA sequence (Fig 1B and C) and confirming the different energy stabilization of the global secondary structure.

**Figure 2:**
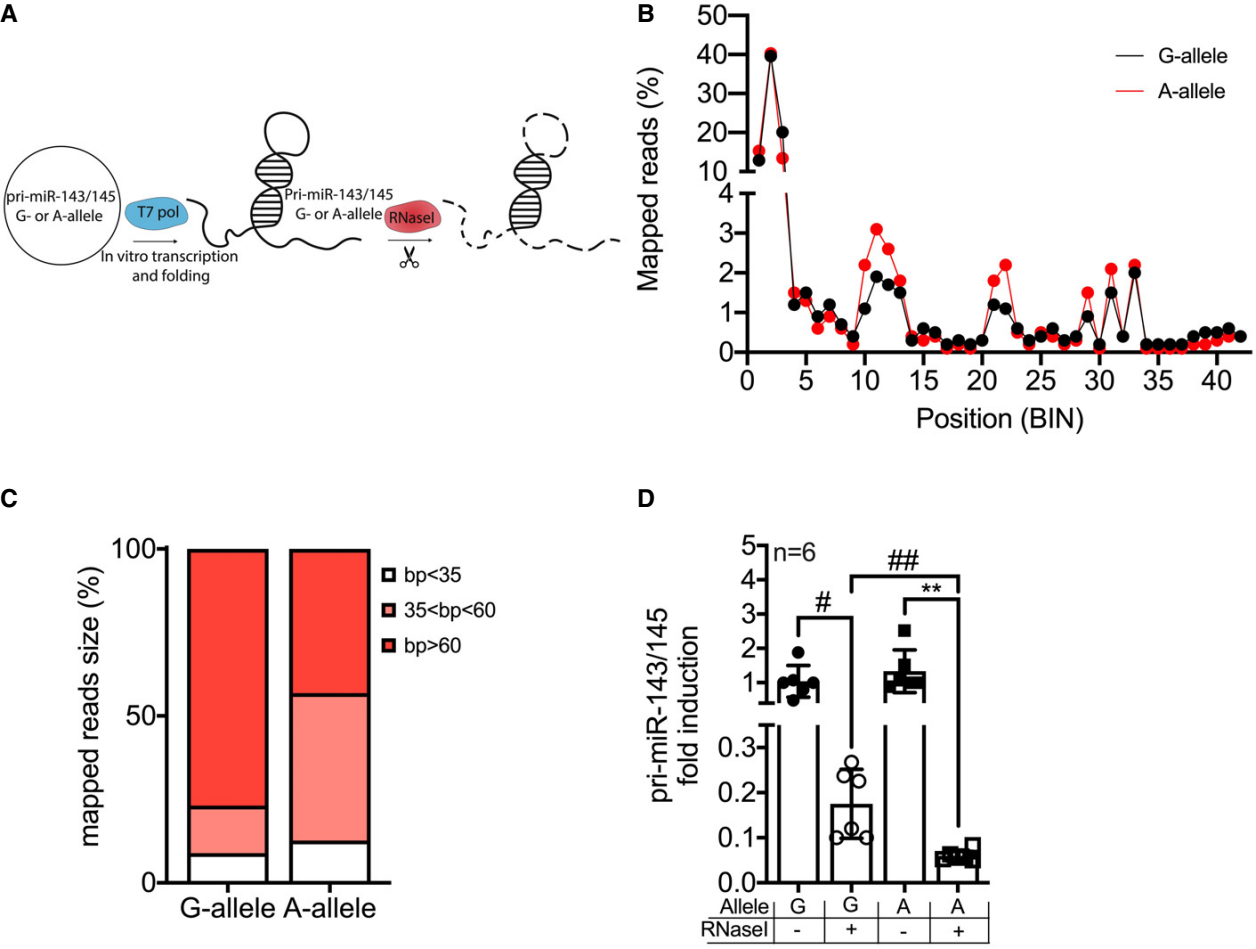
Analysis of the secondary structure of the pri‐miR‐143/145 carrying the G‐ or A‐allele ACartoon showing the experimental setting for the RNaseI cleavage experiments.BMapped reads (%) of RNaseI‐digested pri‐miR‐143/145 carrying G‐ (black line) or A‐allele (red line). The length of the primary miRNA was divided into blocks of 50 bp, defined as BIN.CSizes of the mapped reads (%) for the RNaseI‐digested pri‐miR‐143/145 carrying G‐ or A‐allele; reads were divided into three groups: blocks < 35 bp (white), blocks > 35 bp and < 60 bp (red), and blocks > 60 bp (black).DQuantification by RT–qPCR of RNaseI‐digested pri‐miR‐143/145 carrying G‐ or A‐allele, and not digested controls (*n* = 6). Data information: Data are shown as mean ± standard deviation (SD) and *n* indicates the number of biological replicates. To compare means, one‐way ANOVA with Tukey's multiple comparisons test was used. ^#^Adj *P* = 0.021, ^##^Adj *P* = 0.041, **Adj *P* = 0.015.

**Figure EV2:**
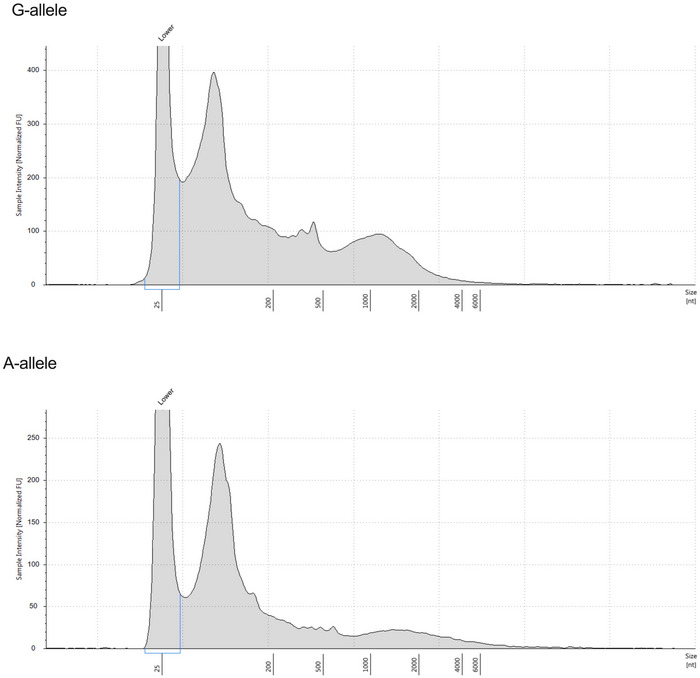
TapeStation profiles of the G‐ and A‐allele digested pri‐miR‐143/145

To corroborate this hypothesis, we evaluated *in vitro* whether there were differences in the structures of the pri‐miRs carrying the WT allele (G) and the *rs41291957* variation (A). To address this, we performed *in vitro* transcription, folding and digestion with RNaseI, an enzyme that degrades linear RNA but not the duplex form (Fig 2A), and then employed TapeStation analysis to assess the sizes of the obtained fragments. We found that the SNP transcript generated an increased number of shorter segments (Fig EV2). Subsequently, the digested RNAs were processed for RNA sequencing, and the obtained reads aligned versus the reference sequences carrying either the WT (G) or the mutated (A) allele. This analysis indicated that the two pri‐miRNAs had different read enrichment profiles (Fig 2B), a finding strongly suggestive of the two RNAs having different secondary structures, as already indicated by the *in silico* modelling (Fig 1B). Furthermore, the presence of *rs41291957* resulted in a higher number of short (bp < 35) and medium (35 > bp < 60) reads, confirming the differential digestion result obtained with RNaseI (Fig 2C). These observations were further validated by quantitative PCR (RT–qPCR) analysis, which clearly demonstrated an increased digestion rate for the *rs41291957* sequence (Fig 2D). Of note, this finding is in line with a less stable secondary structure, as suggested by the thermodynamic profile (Fig EV1A and B).

Altogether, modelling and *in vitro* results supported the hypothesis that the A‐allele facilitates the processing of miR‐143 and miR‐145 transcripts.

### miR‐SNP *rs41291957* and miR‐143/145 processing *in vitro*

**Figure 3:**
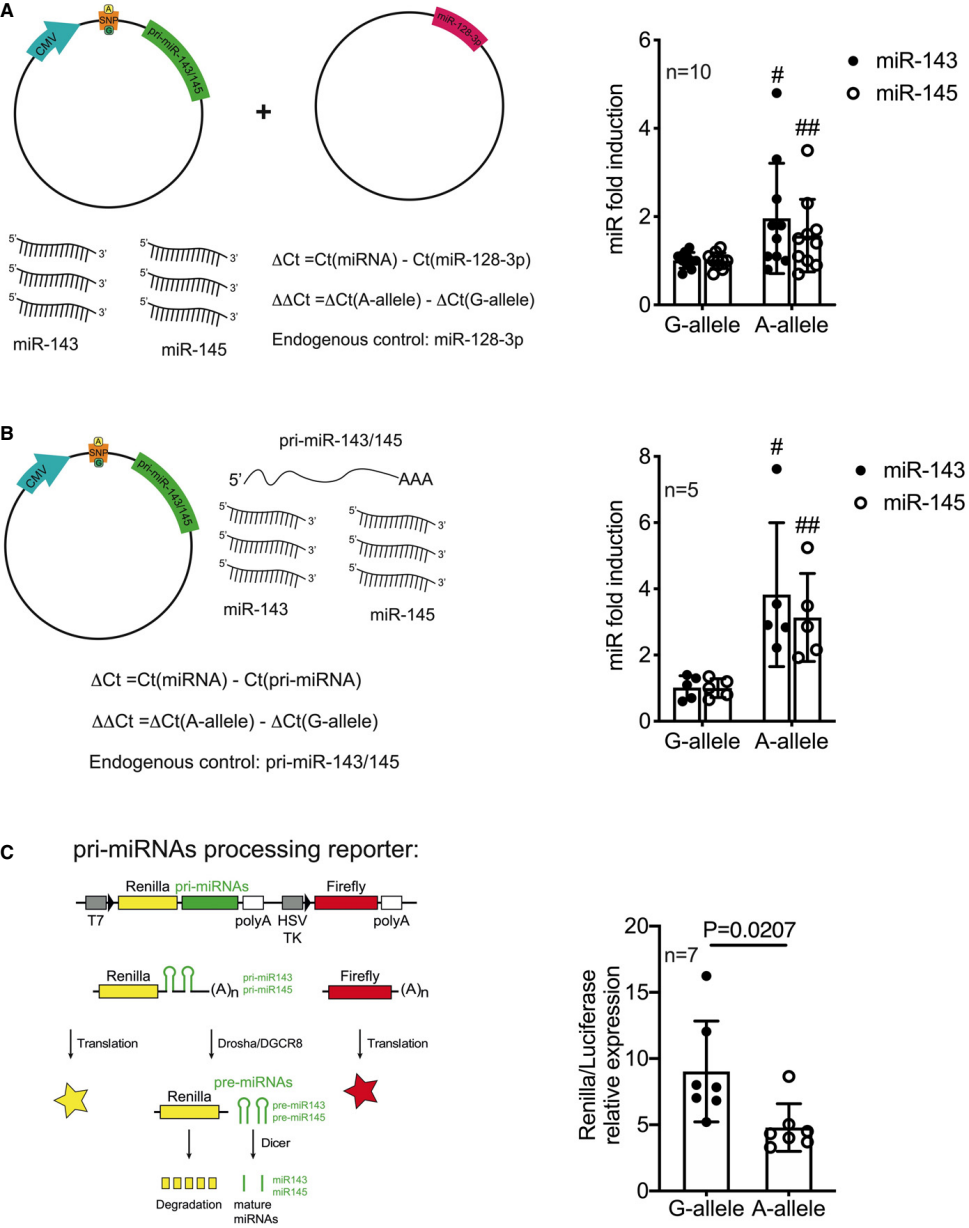
Mature miR‐143 and miR‐145 levels are increased by the presence of the A‐allele ACartoon showing the method utilized to quantify miR‐143 and miR‐145 levels in the presence of the A‐allele (left). Mature miRNA level quantification (RT–qPCR) in HEK‐293T cells transfected with vectors carrying the G‐ or A‐allele and normalized on miR‐128 (*n* = 10; right).BCartoon showing the method utilized to quantify miR‐143 and miR‐145 maturation levels in the presence of the A‐allele (left). Mature miRNA level quantification (RT–qPCR) in HEK‐293T cells transfected with vectors carrying the G‐ or A‐allele and normalized on pri‐miR‐143/145 (*n* = 5; right).CCartoon showing the reporter construct method utilized to quantify miR‐143 and miR‐145 processing (left). Quantification in HCASMCs transfected with vectors carrying the pri‐miR‐143/145 as 3′UTR G‐ or A‐allele and normalized on Luciferase signal (*n* = 7; right). Data information: Data are shown as mean ± standard deviation (SD), and *n* indicates the number of biological replicates. To compare means, unpaired Student’s *t*‐test was used, considering data from G‐allele as control; For A: ^#^ *P* = 0.0286, ^##^ *P* = 0.0456; For B: ^#^ *P* = 0.004, ^##^ *P* = 0.000006.

To assess whether the presence of *rs41291957* affects miR‐143/145 abundancy, we cloned the primary bicistronic miR‐143/145 transcript in an expression vector, using the reference WT sequence, and then introduced the *rs41291957* minor‐A‐allele by directed mutagenesis (Appendix Fig S1A). The constructs containing either the reference WT sequence (G) or the *rs41291957* variant (A) were then transfected into HEK‐293T cells—which express endogenous miR‐143/145 at a low basal level—together with a plasmid carrying the mature sequence of an unrelated miRNA (i.e., miR‐128) in order to normalize the effects of transfection. We found that the presence of the A‐allele was associated with significantly increased levels of mature miR‐143 and miR‐145 (Fig 3A).

We then assessed whether the variation might influence miRNA processing. We reasoned that since the G and A vectors differed only in the variation sequence and that transcription was guided by the cytomegalovirus (CMV) promoter, pri‐miR‐143/145 expression should not differ between the two plasmids; any difference in mature miR‐143 and ‐145 levels should, thus, depend only on miRNA processing. To test this hypothesis, the above‐mentioned constructs (for the G‐ and A‐alleles) were individually transfected in HEK‐293T cells, and mature miR‐143 and miR‐145 levels measured 48 h later by RT–qPCR and normalized on the pri‐miR‐143/145 (Fig 3B, left). The presence of the A‐allele was associated with significantly increased levels of mature miR‐143 and miR‐145 (Fig 3B, right). This finding was strongly indicative of increased processing of the immature miRNA harbouring the SNP.

To further corroborate the processing hypothesis, we utilized a ratiometric dual‐reporter construct (Psicheck2) that simultaneously expressed two chemiluminescent proteins, Renilla and Luciferase, driven by independent promoters (Fig 3C, left). In this reporter, the pri‐miRNA sequences—either WT or SNP—were inserted into the 3′‐UTR of the Renilla expression cassette, whereas the Luciferase gene expression remained unaltered. Thus, these constructs expressed a Renilla–pri‐miRNA fusion RNA. Cleavage of the fused pri‐miRNA by Drosha was expected to affect 3′UTRs, resulting in the removal of the polyadenylation tail, and consequentially leading to the degradation of Renilla mRNA. The luciferase signal allowed for the normalization of changes of individual cell transcriptional and translational activities. Thus, the Renilla/Luciferase firefly ratio negatively correlated with pri‐miRNA processing activity. Both constructs were transfected in WT human coronary artery SMCs (HCASMCs), and indeed, the presence of the A‐allele was associated with a reduced Renilla/Luciferase ratio, confirming the positive influence of this genetic variation on maturation of miR‐143 and miR‐145 (Fig 3C, right).

**Figure 4:**
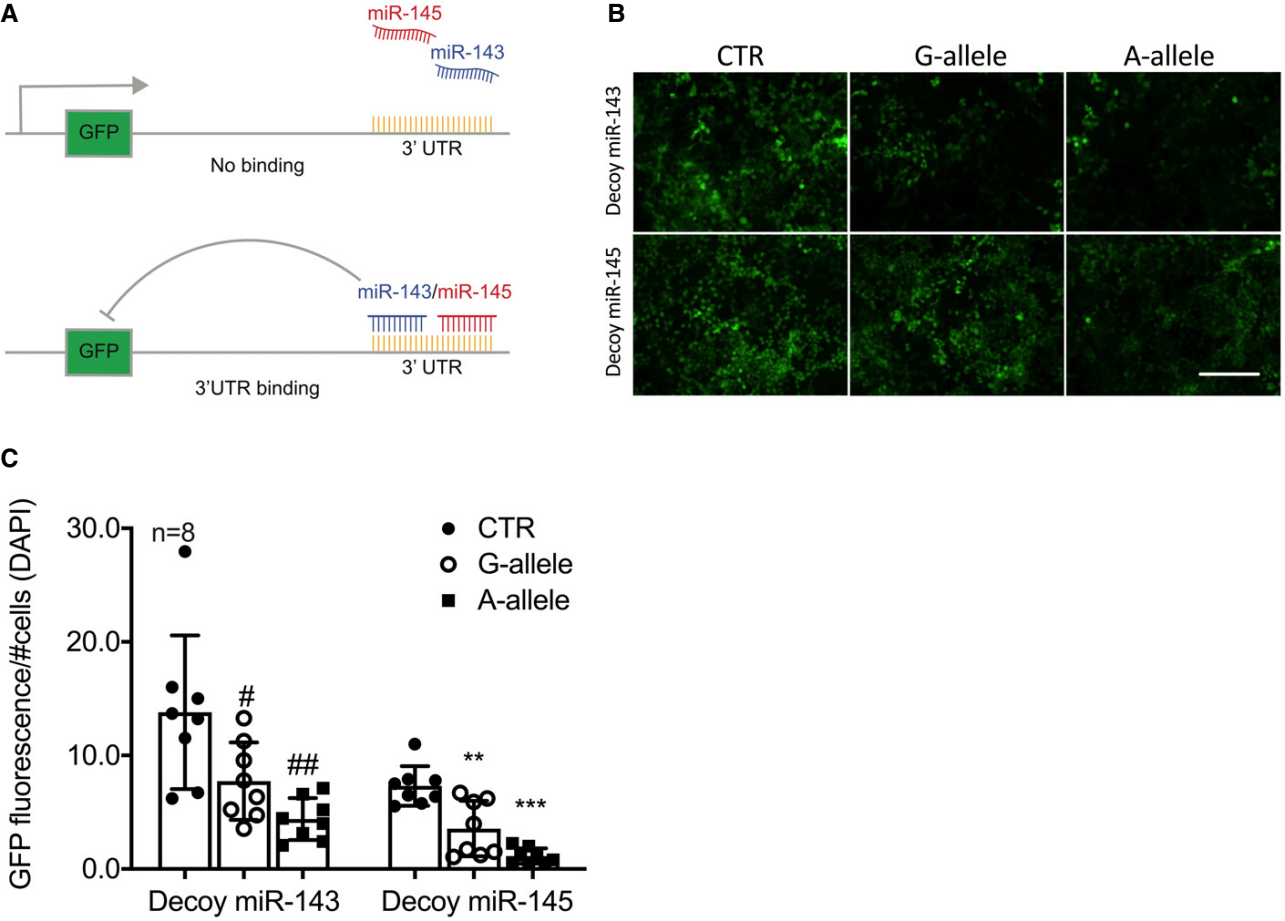
Functionality studies of the A‐allele in cells ACartoon showing the mechanism of action of the utilized decoy system.BHEK‐293T cells transduced with lentiviral particles harbouring either the miR‐143 or miR‐145 decoy sequence were transfected with empty (CTR), G‐allele, or A‐allele vectors. Images were taken 48‐h post‐transfection. Scale bar: 100 μm.CRelative quantification normalized on the number of cells (DAPI signal) (*n* = 8). Data information: Data are shown as mean ± standard deviation (SD), and *n* indicates the number of biological replicates. To compare means, one‐way ANOVA with Tukey's multiple comparisons test was used. ^#^Adj *P* = 0.031, ^##^Adj *P* = 0.047, **Adj *P* = 0.048, ***P = 0.026.

To strengthen the validity of these findings, we generated HEK‐293T cells that constitutively expressed decoy vectors in which green fluorescence protein (GFP) cDNA was linked 3′ to two complimentary tandem sequences containing the miRNA binding sites (Fig 4A and Table EV1), so that when either miR‐143 or miR‐145 bound the mimetic target, GFP was degraded and the fluorescent signal reduced (Climent *et al*, 2015). miR‐143 and miR‐145 decoy‐expressing cells—previously selected by FACS—were transduced with the above‐utilized vectors carrying the G‐ or A‐allele, and GFP intensity evaluated by microscopy; the resulting signal was then normalized for the number of cells (assessed with DAPI staining). We observed a strong reduction in total GFP signal in the presence of the reference allele compared to control vectors (Empty and G‐allele vectors). As expected, further GFP decay was measured in the A‐allele‐transduced population, supporting the functional capacity of this genetic variant to enhance the production of mature miR‐143 and miR‐145 (Fig 4B and Appendix Fig S2A). These findings confirmed that the total fluorescent signal of transduced cells was proportional to miRNA levels: CTR vector > G‐allele vector > A‐allele vector (Fig 4C).

Altogether, these results strongly supported the hypothesis that *rs41291957* minor‐A‐allele aids miR‐143 and miR‐145 expression by increasing pri‐miRNA processing.

### Insertion of miR‐SNP *rs41291957* in CRISPR‐Cas9‐edited HEK‐293T cells increases miR‐143 and miR‐145 transcripts

**Figure 5:**
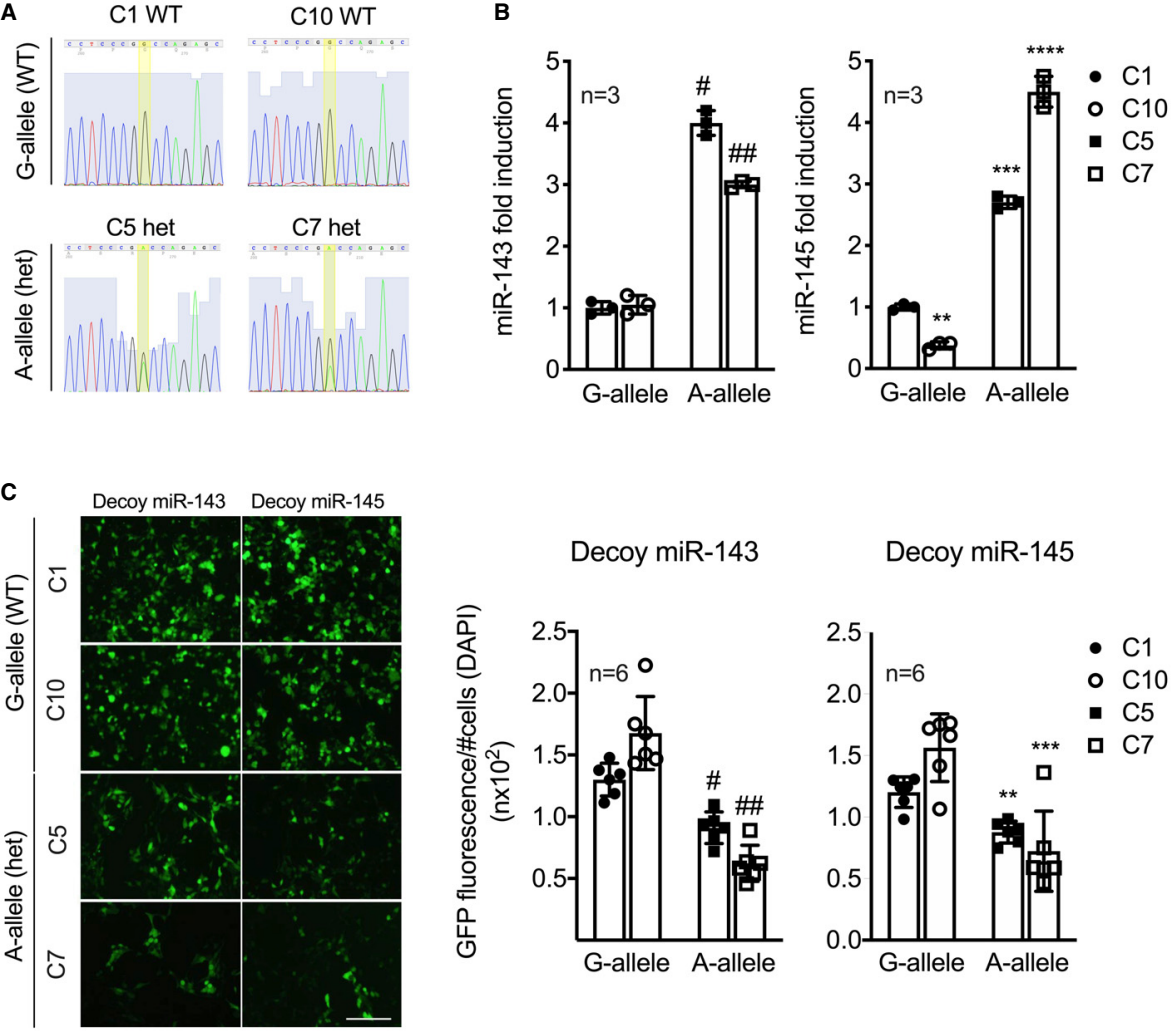
Genetically modified HEK‐293T cells carrying the *rs41291957* variant and effect on miR‐143/145 levels ASanger sequence profiles of the CRISPR/Cas9‐mutated clones.BLevels of miR‐143 and miR‐145 measured by RT–qPCR (*n* = 3).CHEK‐293T clones were transduced with decoy miR‐143 and miR‐145 constructs. Then, 48‐h post‐infection, images were taken and processed: the GFP signal was quantified and normalized by the number of cells (DAPI) (*n* = 6). Scale bar: 50 μm. Data information: Measurements were calculated as per cent of control (C1) as reference. Data are shown as mean ± standard deviation (SD), and *n* indicates the number of biological replicates. To compare means, one‐way ANOVA with Dunnett's multiple comparisons test was used in B and C. For B: ^#^Adj *P* = 7 x 10^−4^, ^##^Adj *P* = 3.6 × 10^−3^, **Adj *P* = 0.017, ***Adj *P* = 5.6 × 10^−4^, ****Adj *P* = 4.7 × 10^−3^; For C: ^#^Adj *P* = 0.011, ^##^Adj *P* = 1.4 × 10^−4^, **Adj *P* = 9.8 × 10^−3^, ***Adj *P* = 0.031. WT, wild‐type clones; het, heterozygote G/A clones.

To further corroborate the findings obtained using constructs transiently carrying *rs41291957*, we adopted a CRISPR‐Cas9 gene‐editing approach based on homologous recombination, obtaining HEK‐293T cells stably carrying the allelic variation. We tested four potential single‐guide (sg)RNAs localized close to the variation site and employed the one with the greatest nicking capacity (Appendix Fig S3). Following transfection, several puromycin‐resistant clones were obtained and subsequently selected, expanded and screened by Sanger sequencing. Through this process, we identified two clones in which the variation was present in heterozygosis (G/A: C5 het; C7 het), whereas two clones with the G‐allele in homozygosis (G/G: C1 wt; C10 wt) were used as controls (Fig 5A). Then, we performed RT–qPCR analysis, normalizing the level of expression of both miRNAs on the endogenous housekeeping gene *U6*. We found increased expression of miR‐143 and miR‐145 in both clones carrying the A‐allele compared to WT ones (Fig 5B), demonstrating that the allelic variation positively modulated miRNA expression.

To validate the biological activity of modulated miR‐143 and miR‐145 expression, the four clones were transiently transfected with decoy constructs for miR‐143 or miR‐145, working as described above (Fig 4A), and the fluorescence signal normalized for the number of (DAPI‐positive) cells 48 h later. According to expectations, clones expressing *rs41291957* had a marked reduction in normalized fluorescence, a finding functionally explaining the increase in endogenous levels of miR‐143 and miR‐145 in the presence of the minor A‐allele (Fig 5C and Appendix Fig S2B).

Taken together, these results showed that miR‐SNP *rs41291957* positively influenced the expression of miR‐143 and miR‐145 in genetically edited cells.

### miR‐SNP *rs41291957* increases the expression of miR‐143 and miR‐145 in HCASMCs, influencing their phenotypic characteristics

**Figure 6:**
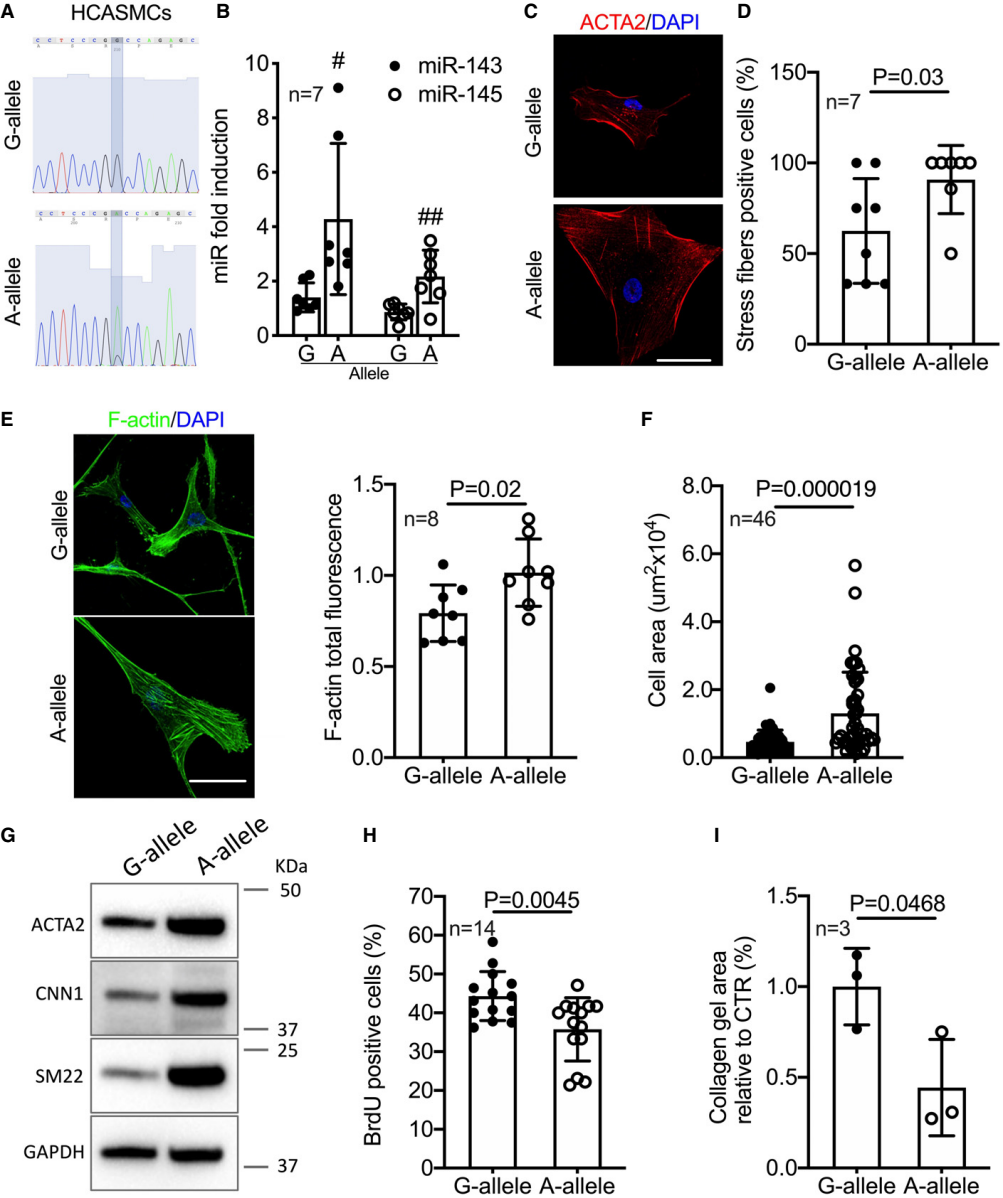
Characteristics of HCASMCs carrying the A‐allele ASanger sequence profiles of the HCASMC primary lines.BLevels of miR‐143 and miR‐145 measured by RT–qPCR (*n* = 7).CRepresentative images of HCASMC clones stained for ACTA2 (representative images chosen for similarity to the global quantification). Scale bar: 10 μm.DQuantification of actin signal fluorescence organized in stress fibres (*n* = 7).ERepresentative images of HCASMC clones stained with phalloidin (representative images chosen for similarity to the global quantification). Scale bar: 10 μm. Quantification of actin signal fluorescence normalized on the total number of cells (*n* = 8).FQuantification of cellular area of HCASMCs carrying the G and A‐allele (*n* = 46).GExpression of human VSMC differentiation markers measured by Western blot for ACTA2, CNN1, and SM22 expression in G‐ and A‐allele cells. Representative images of at least three individual experiments.HProliferation rate measured by BrdU incorporation assay (*n* = 14).IGelatin contraction assay of HCASMCs carrying the G and A‐allele, stimulated with angiotensin II (*n* = 3). Data information: Data are shown as mean ± standard deviation (SD) and *n* indicates the number of biological replicates. To compare means, unpaired Student’s *t*‐test was used considering data from G‐allele as control. For B: ^#^ *P* = 0.01, ^##^ *P* = 0.002. Source data are available online for this figure.

Thereafter, we investigated the impact of this genetic variant in primary HCASMCs. For this purpose, we took advantage of a previous genetic screening performed at the Quertermous laboratory (Liu *et al*, 2018). Among the reported lines, we identified two commercially available lots carrying the reference (G/G) or the mutated (G/A) *rs41291957* allele obtainable from the same vendor (Fig 6A and Appendix Fig S4A). RNAs from both clones were collected at different passages and the levels of mature miR‐143 and miR‐145 measured by RT–qPCR. Data clearly showed that the A‐allele positively modulated miR‐143 and miR‐145 expression in HCASMCs (Fig 6B). Of note, cells carrying the A‐allele appeared phenotypically different from the control (G/G) ones. Indeed, they were wider and had a rearranged cytoskeleton (Fig 6C and Appendix Fig S4B), as demonstrated by an increase in the number of cells presenting highly organized smooth muscle actin (ACTA2) stress fibres (Fig 6D), findings suggestive of a more differentiated state.

**Figure EV3:**
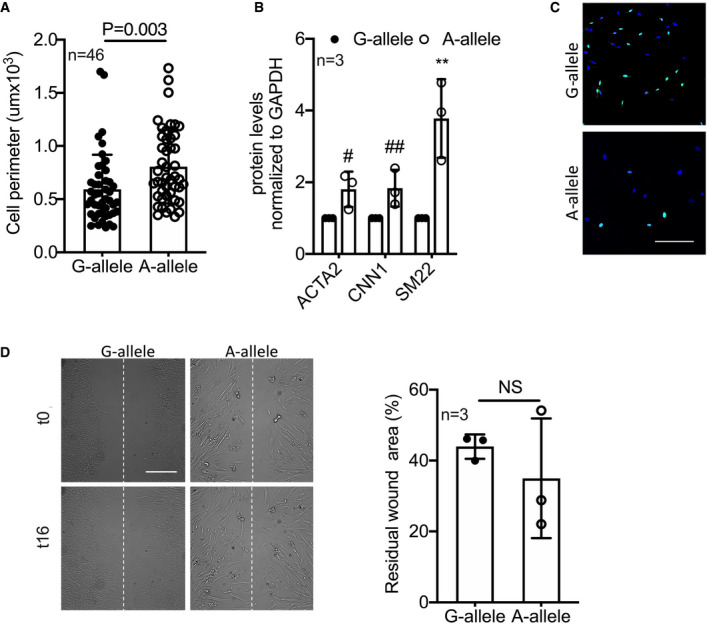
HCASMC biological feature analysis AQuantification of the perimeter of HCASMCs carrying the G‐ and A‐allele (*n* = 46).BQuantification of Western blots in Fig 6G (*n* = 3).CRepresentative picture of BrdU incorporating HCASMCs. Scale bar: 50 μm.DMigration properties measured by scratch (*n* = 3). Scale bar 250 μm. Data information: Data are shown as mean ± standard deviation (SD), and *n* indicates the number of biological replicates. To compare means, unpaired Student’s *t*‐test was used considering data from G‐allele as control. For B: ^#^ *P* = 0.0048, ^##^ *P* = 0.0047, **P = 0.011. NS: not statistically significant.

To address this in detail, we evaluated F‐actin organization (Fig 6E), finding that miR‐SNP *rs41291957* promoted F‐actin organization and increased the area (Fig 6F) and external dimensions (Fig EV3A) of the cells. At the translational level, increased expression of *ACTA2*, *CNN1* and *SM22*—markers of VSMC differentiation—was observed (Figs 6G and EV3B). Coherently, in the presence of this polymorphism there was less proliferation (Figs 6H and EV3C) and increased contractility upon stimulation with Angiotensin II (AngII) (Fig 6I), but no difference in migration (Fig EV3D).

Collectively, these results clearly suggested that the A‐allelic variant promoted a differentiated phenotype in human VSMCs by modulating expression of miR‐143 and miR‐145.

### Inhibition of miRNA‐143 and miR‐145 blunts miR‐SNP *rs41291957‐*mediated HCASMC phenotype changes

**Figure 7:**
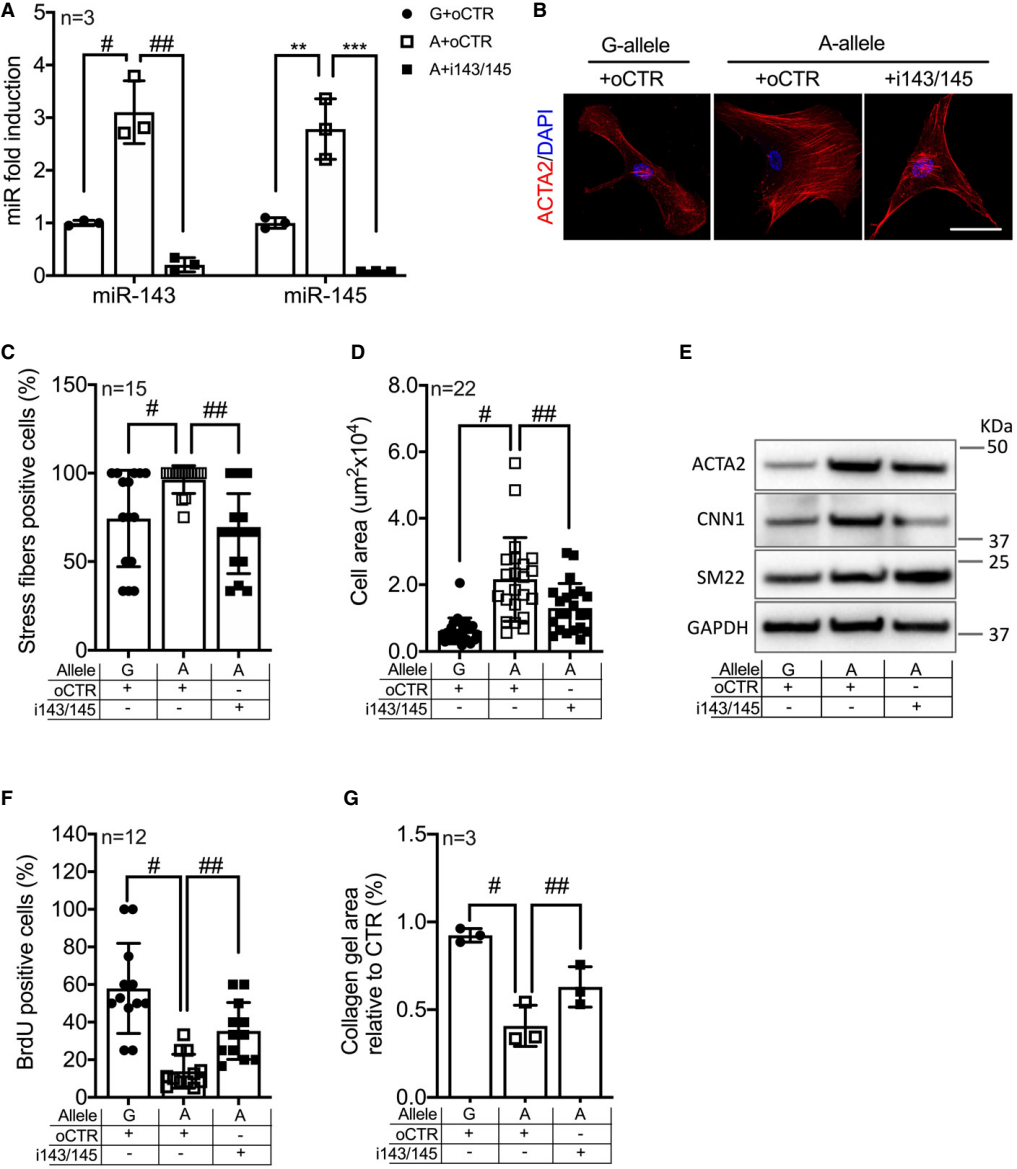
miRNA‐143/145 silencing disrupts the effect of miR‐SNP *rs41291957* in HCASMCs ALevels of miR‐143 and miR‐145 measured by RT–qPCR in the rescue setting (*n* = 3).BRepresentative images of HCASMC clones stained for ACTA2 (representative images chosen for similarity to the global quantification). Scale bar: 10 μm.CQuantification of actin signal fluorescence organized in stress fibres in the rescue setting (*n* = 15).DQuantification of cellular area of HCASMCs in the rescue setting (*n* = 22).EWestern blot for ACTA2, CNN1 and SM22 expression in the rescue setting. Representative images of at least three individual experiments.FProliferation assay measured by BrdU incorporation in the rescue setting (*n* = 12).GGelatin contraction assay of HCASMCs stimulated with angiotensin II in the rescue setting (*n* = 3). Data information: Data are shown as mean ± standard deviation (SD), and *n* indicates the number of biological replicates. To compare means, one‐way ANOVA with Tukey’s multiple comparisons test was used. For A: ^#^Adj *P* = 0.036, ^##^Adj *P* = 0.006, **Adj *P* = 0.049, ***Adj *P* = 0.017; For C: ^#^ *P* = 0.037, ^##^ *P* = 0.0003; For D: ^#^ *P* = 0.0001, ^##^ *P* = 0.033; For F: ^#^ *P* = 0.034, ^##^ *P* = 0.015; For G: ^#^ *P* = 0.0002, ^##^ *P* = 0.01. Source data are available online for this figure.

**Figure EV4:**
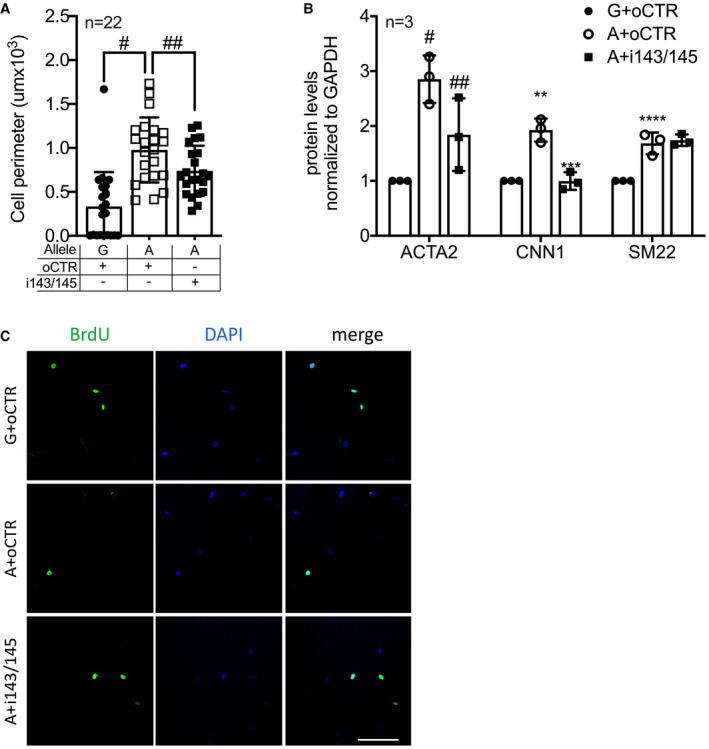
miR‐143 and miR‐145 inhibition affects HCASMCs biological features AQuantification of size of HCASMCs carrying either the G‐ or A‐allele transfected with a control oligonucleotide (oCTR) or after miR‐143/145 inhibition (i143/145) (*n* = 22).BQuantification of Western blots in Fig 7E (*n* = 3).CRepresentative picture of BrdU incorporation. Scale bar: 50 μm. Data information: Data are shown as mean ± standard deviation (SD), and *n* indicates the number of biological replicates. To compare means, one‐way ANOVA with Tukey’s multiple comparisons test was used. For A: ^#^Adj *P* = 0.0001, ^##^Adj *P* = 0.0475; For B: ^#^Adj *P* = 0.032, ^##^Adj *P* = 0.033, **Adj *P* = 0.03, ***Adj *P* = 0.025, ****Adj *P* = 0.048.

**Figure EV5:**
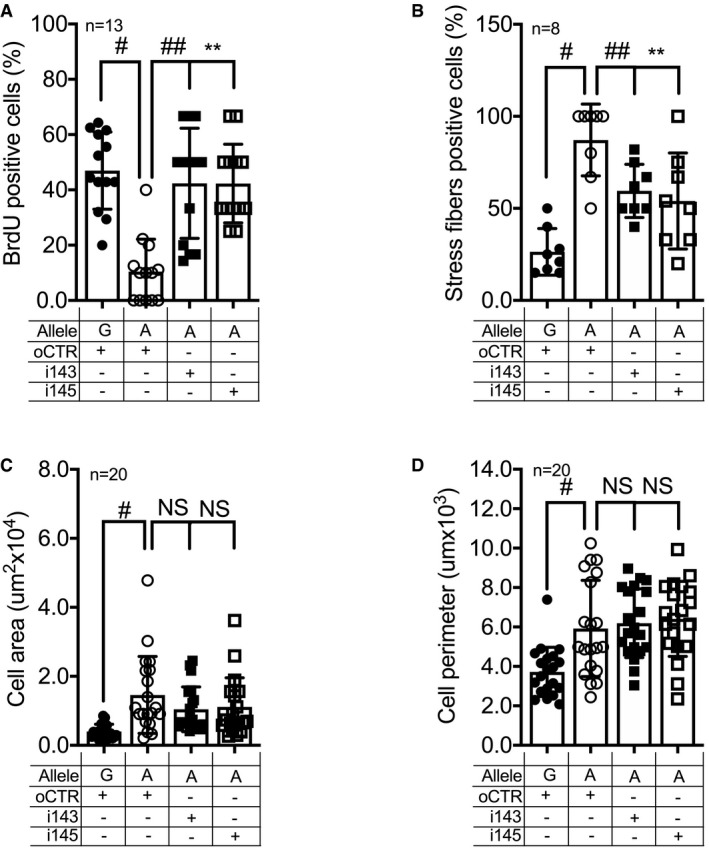
miRNA‐143 or 145 silencing partially rescues the effects of miR‐SNP *rs41291957* on HCASMCs AProliferation assay measured by BrdU incorporation in the rescue setting (*n* = 13).BQuantification of actin signal fluorescence organized in stress fibres in the rescue setting (*n* = 8).CQuantification of cellular area of HCASMCs in the rescue setting (*n* = 20).DQuantification of cellular perimeter of HCASMCs in the rescue setting (*n* = 20). Data information: Data are shown as mean ± standard deviation (SD), and *n* indicates the number of biological replicates. To compare means, one‐way ANOVA with Tukey's multiple comparisons test was used. For A: ^#^Adj *P* = 0.0001, ^##^Adj *P* = 0.0026, **Adj *P* = 0.0001; For B: ^#^Adj *P* = 0.0001, ^##^Adj *P* = 0.039, **Adj *P* = 0.011; For C: ^#^ *P* = 0.0015; For D: ^#^ *P* = 0.0026. NS: not statistically significant.

“Phenotypic rescue” experiments combining the use of two locked‐nucleotide anti‐miRs (LNAs) against both miRNAs (i143+i145) were carried out to demonstrate that the differentiated/contractile phenotype of A‐allele‐carrying HCASMCs (A‐HCASMCs) was dependent on miR‐143 and miR‐145 expression. First, RT–qPCR analysis confirmed the reduction of miR‐143 and miR‐145 in cells transduced with the anti‐miRNA oligonucleotides compared to control oligonucleotide‐transfected cells (Fig 7A). Normalization of miRNA expression blunted the biological effects of *rs41291957* in mutant A‐HCASMCs, re‐establishing the features of the G‐allele‐carrying cells (G‐HCASMCs). This was already evident when observing ACTA2 organization in A‐HCASMCs transfected with the anti‐miRNA oligonucleotides (Fig 7B): Indeed, the percentage of stress fibres (Fig 7C) and the sizes (Figs 7D and EV4A) of these cells were comparable with those of G‐HCASMCs. From the molecular point of view, i143/145 A‐HCASMCs had normalized protein levels of ACTA2 and CNN1, but not SM22, which probably follows different kinetics (Figs 7E and EV4B). The findings strongly indicated it was possible to reinstate the differentiated state of WT HCASMCs by solely restoring the expression of the two miRNAs. Assessments of proliferation (Figs 7F and EV4C) and contractility (Fig 7G) confirmed this hypothesis. In contrast, inhibition of a single miRNA family member resulted in incomplete recovery of the features observed in HCASMCs carrying the G‐allele (Fig EV5). Thus, the impact of the genetic variant on VSMCs relies on both members of miRNA143/145 cluster rather than on a single miRNA.

Altogether, these findings proved that the effects of miR‐SNP *rs41291957* on phenotypic switching in human VSMCs were directly dependent upon modulation of miRNA‐143/145 cluster expression.

### miR‐SNP *rs41291957* is protective for chronic total occlusion in CAD patients

High levels of expression of miR‐143 and miR‐145 are associated with a functional vascular phenotype (Boettger *et al*, 2009; Cheng *et al*, 2009; Cordes *et al*, 2009; Xin *et al*, 2009; Quintavalle *et al*, 2010; Lovren *et al*, 2012; Climent *et al*, 2015). Therefore, to assess the clinical relevance of the data obtained *in vitro*, we screened for *rs41291957* in patients with stable CAD (selected and enrolled from the Neapolis and LURIC studies). Clinical and angiographic features of all patients and of patients stratified for *rs41291957* genotypes are given in Tables EV2 and EV3.

**Figure 8:**
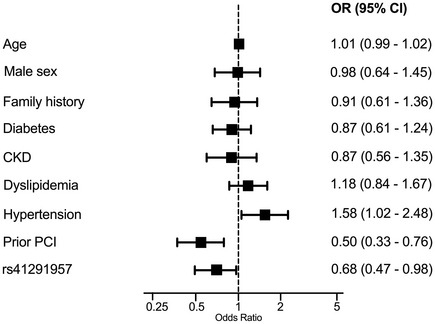
Association of chronic total occlusion with clinical and angiographic features in the Neapolis cohort Age is presented per 1 year/point increase. CKD = chronic kidney disease, CI = confidence interval, OR = odds ratio, PCI = percutaneous coronary intervention.

In the Neapolis cohort, 18.9% of patients carried the *rs41291957* A‐allele (AA+GA): this subgroup was associated with a significant reduction in the prevalence of CTO compared to patients homozygous for the G‐allele, despite the higher rate of concomitant diabetes (the correlation with diabetes, which was nominally significant, did not survive multiple‐testing correction). Furthermore, stratification of the Neapolis cohort for CTO revealed that those with this type of lesion had significantly more complex, bifurcated and thrombotic lesions together with an increased length of the applied stents (Table EV4). Upon multivariate analysis, *rs41291957* variation (from GG to GA or AA), hypertension, and prior percutaneous coronary intervention were independently associated with the presence of CTO (Fig 8).

Consistent with these data, 20.4% of LURIC‐cohort patients carried the *rs41291957* A‐allele (AA+GA). Of note, this subgroup had significantly lower frequencies of previous and familial cases of MI and a lower prevalence of dyslipidaemia compared to patients with G‐allele homozygosis. Furthermore, *rs41291957* was found to be significant in a multivariate analysis of CAD status adjusted for age, sex, type 2 diabetes, family history of MI, dyslipidaemia and hypertension (Table 1).

**Table 1:** Multivariate association results for *rs41291957* in LURIC study CAD patients.

CHR	Pos	SNP	A1	*N*	OR	95% CI	*P* value	Phenotype
5	148808390	*rs41291957*	A	2,601	0.78	0.62–0.97	0.03	CAD

A1, Allele; CHR, chromosome; *N*, number of analysed individuals; OR, odds ratio; Pos, genomic position.

Collectively, these findings demonstrated that the A‐allele was directly associated with reduction of CAD events.

## Discussion

In this study, we have determined a link between disease progression in stable CAD patients and a VSMC‐specific miR‐SNP—*rs41291957*—that affects miR‐143 and miR‐145 expression.

Following vascular injury, VSMCs switch from a contractile to a proliferative phenotype, a phenomenon associated with reduced levels of miR‐143 and miR‐145. This occurs via a complex molecular network involving the PDGF, TGF‐β and Notch pathways (Cheng *et al*, 2009; Elia *et al*, 2009; Boucher *et al*, 2011). Vascular delivery of both miRNAs has been reported to strongly reduce the development of vascular diseases, including atherosclerosis (Lovren *et al*, 2012) and stenosis (Cheng *et al*, 2009; Elia *et al*, 2009). The reasons for such remarkable effects might be several: upregulation of different direct targets involved in VSMC migratory and proliferative capacities (Quintavalle *et al*, 2010; Elia *et al*, 2018); modulation of cell types other than VSMCs, including endothelial cells (Hergenreider *et al*, 2012; Sala *et al*, 2014; Climent *et al*, 2015); and modulation of other non‐coding RNAs favouring neointimal formation (Hall *et al*, 2019; Farina *et al*, 2020).

Previous studies have assessed association between *rs41291957* and disease outcome. No correlation was found in a Chinese population with sporadic congenital heart disease (Yang *et al*, 2014), but a significant correlation was observed for this SNP and cancer (Li *et al*, 2013; Wu *et al*, 2016), with *rs41291957* speculated to be involved in regulating the activity of the miR‐143/145 promoter. However, since the promoter is located at a significant distance from the miR‐143/145 locus, a *cis* effect on the promoter is highly unlikely (Cordes *et al*, 2009; Xin *et al*, 2009). In contrast, we demonstrate here that the A‐allele variation modulates miR‐143/145 cluster maturation. A seminal study by Carlo M. Croce’s team showed that a single genetic variation in pri‐miR‐16‐1 bears functional consequences on miRNA maturation, increasing cancer susceptibility (Calin *et al*, 2005). Similarly, we determined that genetically modified cells as well as primary VSMCs carrying the *rs41291957* variant had increased expression levels of miR‐143 and miR‐145, which in turn triggered a differentiated/contractile cellular phenotype.

Of importance for the clinical setting, we found that miR‐SNP *rs41291957* was associated with the presence of a specific CAD phenotype in two large populations of stable CAD patients originating from different geographic areas and, therefore, of different ancestral origins. In the southern European Neapolis cohort, the analysed SNP protected against CTO development. CTO is defined as complete or nearly complete obstruction of a coronary artery, present for at least 3 months (Fraga *et al*, 2005). Whereas in the acute setting occlusion of collateral vessels develops suddenly, and adequate blood requirements may not be able to be restored, in CTO they develop gradually, limiting ischaemia and symptoms. From the etiological point of view, coronary CTO is characterized by the presence within the artery of atherosclerotic plaques in which VSMC dysfunction closely associates with the recanalization outcome (Brugaletta *et al*, 2012). In this cohort, we also observed a nominal association of *rs41291957* with diabetes, which did not survive multiple‐testing correction, suggesting its independence to vascular biology. Of note, the CTO association was indeed independent from other potential confounders, including diabetes, as indicated by multivariate analysis. Unfortunately, in the LURIC cohort—utilized to validate the Neapolis findings—data on CTO were unavailable. Nonetheless, using a more stringent statistical method, we were still able to correlate *rs41291957* with the general CAD phenotype.

Some limitations of our study should be underlined. First, we were not able to measure circulating miRNAs because plasma samples were not available; thus, future prospective investigations are required to compare miRNAs levels in the blood with *rs41291957* genotype. Secondly, the retrospective analysis of the clinical studies did not allow us to discover any causal relationships between the A‐allele and the development of CAD/CTO. However, the *in silico* and *in vitro* findings were strongly coherent with the mechanistic explanation of this association and strongly support the hypothesis of a causal relationship. Finally, despite our extended experience with animal models, including miR‐143/145 knockouts, we did not perform any *in vivo* experiments. Indeed, we believe that knock‐in animals carrying the variation—besides being complex to generate—would very unlikely phenocopy what we observed in human VSMCs due to the sophistication of human genetic/epigenetic regulation.

In conclusion, findings of the present study indicate that *rs41291957* modulates miR‐143 and miR‐145 expression, influencing the risk of CAD. Although future studies are warranted, this study is suggestive of miR‐SNP *rs41291957* being a useful marker for the prognosis of CAD.

## Materials and Methods

### Selection of miR‐SNPs

The Ensembl database was systematically searched. SNPs affecting the miR‐143/145 cluster that could also improve detection of frequency differences in CAD patients and that had increased probability of affecting mature miRNA levels were identified according to the following inclusion criteria: variation lying within the primary‐miR‐143/145 transcript, with a minor allele frequency (MAF) > 10%.

### Clinical study population

We studied two independent cohorts of CAD patients in order to determine the relevance of identified SNPs for CAD pathogenesis.

#### Derivation cohort

Initial analysis was performed utilizing the Neapolis study. It describes a cohort of 1,726 consecutive patients, mostly of southern European (Mediterranean) origin, with stable CAD treated with percutaneous coronary intervention (PCI) between 2010 and 2012 and evaluated retrospectively. A detailed description of enrolment and exclusion/inclusion criteria has been previously published (Viviani Anselmi *et al*, 2013). Briefly, all consecutive patients scheduled for elective PCI with drug‐eluting stents at Clinica Mediterranea (Naples, Italy) were considered for inclusion.

#### Validation cohort

The Ludwigshafen Risk and Cardiovascular Health (LURIC) study served as a validation cohort. It is a prospective cohort of 2,601 individuals of German ancestry, recruited between 1997 and 2000, with characteristics similar to that of the Neapolis study (i.e., stable CAD treated with PCI). A detailed description is published elsewhere (Winkelmann *et al*, 2001).

The studies comply with the Declaration of Helsinki, were approved by the local ethics committees and the experiments also conformed the Department of Health and Human Services Belmont Report. All patients provided informed consent.

### Assessment of CAD phenotype

For the Neapolis cohort, coronary angiograms were reviewed to assess the number of diseased coronary vessels as well as the presence of CTO, bifurcation lesions, calcified lesions, B2/C coronary lesions (as per the AHA/ACC classification) and thrombotic lesions. CTO was defined as the presence of thrombolysis in MI flow within the occluded segment, and angiographic or clinical evidence or high likelihood of occlusion duration ≥ 3 months. A bifurcation lesion was defined as coronary artery narrowing > 1.5 mm in diameter adjacent to a side branch. Intraluminal thrombus was defined as intraluminal globular filling defects in multiple angiographic views.

In the LURIC cohort, CAD was diagnosed with angiograms, calculating vessel stenosis according to several thresholds: 10%, 20%, and 50% in one, two, or three vessels. For this study, a 50% threshold was used (Winkelmann *et al*, 2001).

### Human samples: DNA extraction and genotyping procedure

For the Neapolis study, 4 ml whole peripheral blood was obtained from all patients at the time of PCI. Genomic DNA was extracted from mononuclear cells with the DNA QIAamp Midi kit (Qiagen Inc., CA, USA) according to the manufacturer’s recommendations, and the DNA stored at −20°C until used. The quantity and quality of the genomic DNA was verified with a NanoDrop spectrophotometer (Thermo Fisher Scientific, MA, USA) before being assayed with the ABI PRISM^®^ 7900HT Sequence Detection System (Life Technologies, CA, USA), according to the manufacturer's protocol. Sample processing was fully automated, using the Freedom EVO^®^150 robotic workstation (Tecan Group Ltd., Männedorf, Switzerland). A custom SNP assay was synthesized by Life Technologies (FW: ACAGGAAACACAGTTGTGAGGAATT; RW: CCAACCTGGCCAGG AGAAG; Reporter_1‐VIC: CCTCCCGACCAGAGC; Reportert_2‐NFQ: CTCCCGGC CAGAGC).

In the LURIC study, blood samples were taken on the day of coronary angiography for posterior analysis of metabolites and genotyping (further details are described elsewhere) (Zewinger *et al*, 2017). For the genotypization of the cohort, the CardioMetabochip (Voight *et al*, 2012) and the OmniExpress chip (Illumina, San Diego, CA, USA) were used.

### Cell culture

HEK‐293T cells were cultured in DMEM (Lonza) supplemented with 10% FBS (Lonza), 2 mM glutamine, 1 mM sodium pyruvate and 100 U/ml penicillin–streptomycin. Primary human VSMCs from coronary artery (HCASMC) were purchased from Cell Applications (Cat. #350‐05a: HCASMC WT lot#1386 and HCASMC SNP lot#1483) and cultured in 231 Medium (Life Technologies) supplemented with SMGS (Life Technologies) and 100 U/ml penicillin–streptomycin. All cultures were maintained in a humidified 5% CO_2_ atmosphere at 37°C.

### Quantitative Real‐Time PCR (RT–qPCR)

For primary miRNA expression, RNA was reverse transcribed with a high‐capacity cDNA archive kit (Life Technologies), and RT–qPCR performed using SYBR Green (Promega). The utilized primers are listed in Table EV1. For mature miRNA expression, RNA was reverse transcribed using miRCURY LNA Universal RT (Qiagen). SYBR Green was used to perform the RT–qPCR for mature miR‐143, miR‐145, using U6 as internal control (Exiqon).

### Isolation of genomic DNA from selected cells

DNA was isolated using the NucleoSpin Tissue kit from Macherey‐Nagel, following the manufacturer's instructions. DNA concentration was measured with a NanoDrop instrument (Thermo Scientific).

### Amplification of the target genomic region by PCR

PCRs were performed using Taq DNA polymerase from Biotools, using the following primers: SNP957‐f TTACCACTTCCAGGCTGATG, SNP957‐r GAGATAGAAACTGGTCTGCC. PCR conditions were: 94°C for 3 min; 35X (94°C for 30 s, 57°C for 20 s, 72°C for 30 s); 72°C for 7 min; followed by 4°C. PCR products were analysed by 1% agarose gel electrophoresis and purified from the gel using the QIAquick PCR Purification Kit (Qiagen) for direct sequencing using SNP957‐f primer.

### RNA secondary structure prediction

Secondary structures of the human miR‐143/145 primary sequence were predicted using the centroid estimator method (Ding *et al*, 2005). Minimum free energy was calculated (http://rna.tbi.univie.ac.at/cgi‐bin/RNAWebSuite/RNAfold.cgi) (Mathews *et al*, 2004; Gruber *et al*, 2008). To run the RNA folding prediction, we used the algorithm default parameters [Checked functions. Fold algorithms and basic options: “minimum free energy (MFE) and partition function,” “avoid isolated base pairs”; Dangling end options: “dangling energies on both sides of a helix in any case”; Energy Parameters: “RNA parameters (Turner model, 2004)”; Other Parameters: “rescale energy parameters to given temperature (C)” set at 37C]. The analysed region started −300 bp from the miR‐143 precursor and ended at the end of the miR‐145 precursor.

### Transcription, folding and digestion of RNA *in vitro*

DNA products of the sequences carrying the G‐ or A‐allele controlled by the T7 promoter were obtained by PCR. *In vitro* transcription was performed following the manufacturer’s protocol of the T7 RNA polymerase (T7 polymerase Roche Cat# 10881767001). Briefly, the same amount of DNA was incubated with 1 mM rNTPs (Promega Cat#P113B), 20 U RNase inhibitor (Promega Cat# N261B) 2 h at 37°C and RNA purified with DirectzolT RNA Miniprep (ZymoResearch # ZYR2052). After, *in vitro* annealing of the secondary structure was done by incubating the same amount of RNA for each sample in duplicate at 70°C for 2 min in 10 mM Tris–HCl pH 7.5, 100 nM NaCl, 1 mM EDTA following a 15 min cool down of the samples at room temperature. For RNA cleavage of the secondary structure obtained, one of the duplicates produced was used as control of the digestion (no enzyme). Then, 5 U of RNaseI (Thermo Scientific Cat# EN0601) were added for the digestion in the Enzyme samples for 30 min at 37°C and inactivated at 100°C for 20 min. RNA was extracted using DirectzolT RNA Miniprep (ZymoResearch # ZYR2052). Finally, to evaluate the pri‐miRNA, cDNA was produced using the same amount of RNaseI‐digested RNAs for all samples using High‐Capacity cDNA RT kit (Applied Biosystems Cat# 4368813) and RT–qPCR performed using SYBR Green (Promega).

### Library preparation and bioinformatics analysis of pri‐miR‐143/145 after cleavage

The libraries were prepared with the SMARTer smRNA‐Seq Kit for Illumina (Takara, Cat# 635031) and sequenced on the Illumina Next Seq550 platform. The raw reads were quality checked with FastQC tool version 0.11.9 (https://www.bioinformatics.babraham.ac.uk/projects/fastqc/) and trimmed with cutadapt tools (http://code.google.com/p/cutadapt/) version 1.18 with command:

cutadapt ‐m 15 ‐u 3 ‐a AAAAAAAAAA input.fastq > output.fastq.

The reads of the SNP sample were mapped against the pri‐miR‐143/145 sequence with A‐allele in 209 pb position; the reads of the WT sample were mapped against the WT sequence. Reads mapping was performed with Bowtie tool version 1.2.3 (Langmead *et al*, 2009) with default options. The two pri‐miR‐143/145 sequences were divided into bins of 50 bases each and the reads that mapped in each of the bins were counted, assigning the reads to the bin where they overlapped for more than 50% of their length, using bedtools coverage tool of BEDTools package version 2.29.0 (Quinlan & Hall, 2010). The number of mapped reads in each bin was normalized, dividing their number by the total number of mapped reads. We performed the analysis of reads length distribution importing the bam files of reads alignment in R software version 4.0.5 (https://www.R‐project.org/) with Rsamtools package version 2.6.0 (https://bioconductor.org/packages/Rsamtools) and counting the number of reads that had a length shorter than 35 bp, a length between 35 and 60 bp, and longer than 60 bp, after removing the duplicated reads from bam files with samtools rmdup function of SAMtools package version 1.6 (Li *et al*, 2009). Figures were generated with PRISMA software version 8.0.

### Gene expression calculations

Data in Fig 2D: To evaluate pri‐miR‐143/145 expression following RNAseI digestion, data of the G‐ and A‐allele sequences treated with the enzyme were compared to the respective (untreated) control. The expression values were calculated with the following formula: ΔCt = Ct (pri‐miR143/145 G‐ or A‐allele – With enzyme) − Ct (pri‐miR143/145 G‐ or A‐allele – No enzyme). Data in Fig 3A: To evaluate miR‐143 and miR‐145 expression in the experiment shown in Fig 3A, we utilized miR‐128 derived from a co‐transfected plasmid. The expression values were calculated with the following formula: ΔCt = Ct (mature miR‐143 or miR‐145) − Ct (miR‐128); ΔΔCt = ΔCt (A‐allele) − ΔCt (G‐allele). Data Fig 3B: To evaluate the effect of the variation on miRNA processing, mature miRNA levels were normalized using the primary miRNA transcript as internal control, and the relative quantities calculated with the following formula: ΔCt = Ct (mature miRNA) − Ct (primary miRNA); ΔΔCt = ΔCt (A‐allele) − ΔCt (G‐allele). Here, primary miR‐143/145 was used as an internal control because vectors carrying the G‐ or A‐allele were driven by the same promoter, so no differences in transcription were expected.

Data in Figs 5, 6, 7: For all RT–qPCR data shown in these figures, mature miR‐143 and miR‐145 levels were normalized on the internal housekeeping gene *U6*, since we measured endogenous miRNAs.

### CRISPR‐Cas9 construct design and clone generation

The human primary miR‐143/145 sequence was amplified from VSMCs. SNP *rs41291957* was introduced using the QuikChange Site‐Directed Mutagenesis Kit, as described by the manufacturer (Stratagene). Sanger sequencing confirmed the introduction of the correct SNP variation.

The pSpCas9(BB)‐2A‐Puro (PX459) plasmid containing the human codon optimized SpCas9 gene with puromycin resistance was obtained from Addgene (#48139) (Ran *et al*, 2013). Using the CRISPR design algorithm (http://crispr.mit.edu), we identified four single‐guide (sg)RNAs located upstream of the genetic variation on the reverse DNA strand. Cloning of sgRNAs (sequences in Table EV1) was done according to Feng Zhang Lab CRISPR plasmid instructions (https://www.addgene.org/crispr/zhang/). The guides were then cloned into the plasmid PX459, and cells transfected with the plasmid together with the modified oligonucleotides containing the A‐allele variation (Table EV1). Guide efficiency was evaluated with the GeneArt Genomic Cleavage Detection Kit (Life Technologies).

### Cell transfection and clone selection

To edit the genomic sequence of HEK‐293T cells, the PX459 vector containing the selected sgRNA (Guide 10) was transfected together with the oligonucleotides for the homologous recombination carrying the A‐allele, using Fugene transfection reagent (Promega). Following 72 h of puromycin treatment, clones were then isolated with a limited dilution approach.

To evaluate modulation of the mature miRNAs by DNA vectors, HEK‐293T cells were cultured in DMEM (Lonza) supplemented with 10% FBS (Lonza), 2 mM glutamine, 1 mM sodium pyruvate and 100 U/ml penicillin–streptomycin and maintained in a humidified 5% CO_2_ atmosphere at 37°C. Cells were seeded in 12‐well plates and transfected with 1–2 μg of plasmid DNA, depending on the assay, using the CaCl_2_ method. 48‐h post‐transfection, cells were collected, and total RNA isolated using PureZOL (Bio‐Rad), according to the manufacturer’s protocol.

To study the functionality of the miRNAs, HEK‐293T cells were cultured and transfected with decoys as explained above and, after 48 h, cells collected to evaluate GFP signal in either PBS/0.1% FBS for FACS analysis or PureZOL (Bio‐Rad) for RNA extraction.

### Primary‐miR‐143/145 construct design

The wild‐type (WT) primary transcript sequence for miR‐143/145 was amplified from human VSMCs and cloned into an expression construct controlled by the cytomegalovirus (CMV) promoter (ViraQuest). Afterwards, the SNP variation was introduced using the Site‐Directed Mutagenesis kit (Agilent) and the correct modification confirmed by Sanger sequencing.

### Cell transfection

Sorted HEK‐293T GFP‐positive cells harbouring decoy sequences were transfected with 500 ng of WT or SNP plasmid, using CaCl_2_ in standard conditions. After 48 h, cells were collected and processed for imaging.

### Generation of lentiviral vectors

Production of Lenti‐Empty (CTR), Lenti‐Decoy‐miR‐143 and Lenti‐Decoy‐miR‐145 was performed as previously described (Climent *et al*, 2015). HEK‐293T cells were transduced with the generated lentiviral particles, and GFP‐positive cells were then sorted on a FACS ARIA (BD Biosciences).

### Generation of decoys for miR‐143 and miR‐145

To study the functionality of miR‐143 and miR‐145, production of Lenti‐Decoy‐miR‐143 and Lenti‐Decoy‐miR‐145 was performed as previously described (Climent *et al*, 2015). Afterwards, HEK‐293T cells were transduced with the lentiviral vectors, GFP‐positive cells sorted using a FACS ARIA (BD Biosciences) and kept in culture for experiments.

### Immunocytochemistry

HCASMCs were seeded on a glass‐coverslips, harvested and then fixed in 4% paraformaldehyde. Cell permeabilization was performed with 0.3% Triton X‐100 on ice for 10 min. Then, samples were blocked in PBS, 0.02% NP‐40, 1% bovine serum albumin (BSA) for 30 min. Smooth muscle actin (ACTA2) was visualized using a monoclonal primary antibody (Abcam, Ca# ab32575). Thereafter, MACH 1 Universal HRP‐Polymer Detection kit (BIOCARE Medical, Ca# M1U539) was utilized, following the manufacturer’s indications. After DAB development, haematoxylin counterstaining was performed, and images acquired with the VS120 DotSlide slide scanner (Olympus).

### Immunostaining

ACTA2 and Phalloidin staining were performed to assess stress fibres and actin organization. HCASMCs were fixed in 4% paraformaldehyde and permeabilized with 0.3% Triton X‐100 at room temperature for 10 min. For ACTA2 staining, cells were marked using a monoclonal primary antibody (Abcam, Ca# ab32575) and a secondary antibody labelled with Alexa‐488; phalloidin staining was performed using a Phalloidin‐Alexa 488 (Life Technologies) primary conjugated antibody. Then, 4',6‐diamidino‐2‐phenylindole (DAPI) counterstaining was utilized to visualize nuclear localization. Finally, Diamond Prolong (Life Technologies) was used as the mounting medium. Confocal acquisitions were obtained using a SP8I Leica spectral confocal laser scanning microscope with a 20× objective lens and analysed with Fiji software. To evaluate actin filaments, smooth muscle actin (ACTA2) was measured as percentage of cells displaying stress fibres over the total number of cells per field. Alternatively, for phalloidin staining we measured the total GFP intensity over the total number of cells in each field.

### BrdU incorporation assay

To evaluate HCASMC proliferation, cells were seeded on a glass coverslip at a 20% confluence and subsequently incubated with 5’‐Bromo‐2’‐Deoxy‐uridine (BrdU) reagent (Life Technologies, Ca# 000103) for 24 h, following the manufacturer’s indications. Cells were then fixed with 4% PFA for 15 min, rinsed, treated with 2 M HCl for 10 min at room temperature, rinsed, treated with Borate Buffer for 10 min at room temperature and then addressed to an immunofluorescence procedure. BrdU staining was performed using a primary anti‐BrdU antibody (Santa Cruz, Ca# sc‐32323) and visualized with a donkey anti‐rabbit Alexa‐488 secondary antibody (Life Technologies, Ca# A‐21202). DAPI counterstain was performed to visualize nuclear localization. Confocal acquisitions were performed as mentioned above. BrdU‐positive cells were detected and counted over total number of cells in each field using Fiji software.

### Wound healing assay

HCASMCs were cultivated in 12‐well plates until they reached 100% confluence. A scratch in the cell layer was produced with a 20 µl pipette. Afterwards, cells were washed with 1× PBS and cell migration evaluated over time using the DMI8 Live Cell system (Leica).

### Functional contractility assay

HCASMC were detached using Trypsin‐EDTA and resuspended at the concentration of 1.2x10^5^ cells/ml. Then, 0.2 ml of cell suspension was added to 0.1 ml of 3 mg/ml collagen solution (08‐115 Sigma) diluted in 0.1% Acetic Acid, and mixed thoroughly in a 1.5 ml Eppendorf tube. After adding an appropriate volume of 1 M NaOH to the mixture of cells and collagen, 200 μl of the mixture was immediately transferred to 48‐well plate. Gels were allowed to solidify for 20 min at room temperature and then 250 μl of fresh complete DMEM added. Thereafter, cells were immediately stimulated with 500 nM of angiotensin II to induce HCASMC contraction. Plates were then transferred to a 37°C incubator with a humidified 5% CO_2_ atmosphere. The extent of gel contraction was measured by calculating the area of gel with ImageJ software.

### Luciferase reporter assay

HCASMCs were transfected in 12‐well dishes with 0.2 µg of WT or SNP primary miR143/145 psiCheck2 reporter. Transfection was performed with Lipofectamine LTX+Plus reagent (Life Technologies), following the manufacturer's protocol. Finally, cells were harvested 48‐h post‐transfection and analysed using the dual‐luciferase reporter assay system (Promega) as described by the manufacturer.

### Western blotting

The utilized antibodies were the following: anti‐ACTA2 (Abcam, Ca# ab32575) at the dilution of 1:2,000, Anti‐CNN1 (Santa Cruz, Ca# sc‐136987) at the dilution of 1:500, Anti‐SM22 (Santa Cruz, Ca# sc‐53932) at the dilution of 1:500, and anti‐GAPDH (Santa Cruz, Ca# sc‐32233) 1:2,000. The secondary antibodies were the following: Goat anti‐Rabbit IgG (H + L) Cross‐Adsorbed Secondary Antibody, HRP (Life Technologies, Ca# G‐21234), Goat anti‐Mouse IgG (H + L) Cross‐Adsorbed Secondary Antibody, HRP (Life Technologies, Ca# G‐21040), at the dilution of 1:5,000.

### Statistical analyses

For data from the Neapolis cohort, normality assumption was verified using the Shapiro–Wilk test. Categorical variables were expressed in percentages. Comparison between groups was performed by Student's *t*‐test or chi‐square test, as appropriate. For genetic analysis, Hardy–Weinberg equilibrium was determined using the chi‐square goodness‐of‐fit test and the average genotyping rate of *rs41291957* was successfully verified for all patients. To evaluate the association between *rs41291957* and CTO, logistic regression analysis under log‐additive genetic model was performed. To adjust the multivariate model, clinical predictors for CTO were previously selected according to the literature and significance (*P* < 0.1) at univariate analysis (Table EV4).

For LURIC, we performed logistic regressions with CAD status as a dependent variable, with adjustment for sex, age, type 2 diabetes, family history of MI, dyslipidemia status, and hypertension, using PLINK (Purcell *et al*, 2007).

To compare means in *in vitro* studies, data were first subject to ROUT test to identify possible outliers; for experiments with more than five biological replicates, data normality was calculated with the Kolmogorov–Smirnov (K‐S) test; statistical analyses were then performed with single or multiple two‐tailed *t*‐test (parametric unpaired or paired, two group of analysis), Mann–Whitney *U‐*test (nonparametric unpaired, two group of analysis) or repeated‐measures analysis of variance (ANOVA) followed by *post hoc* Tukey or Dunnett's multiple comparisons test for pairwise comparisons. Each experiment was replicated at least three times and the exact number or replicates reported in each figure panel. Data are shown as mean ± standard deviation (SD), unless differently noted. A 2‐tailed *P*‐value < 0.05 was deemed as statistically significant.

Statistical analyses were performed with the Stata v.11/SE program (College Station) and PRISM software (GraphPad).

## Author contributions

IFH and MC conducted experiments, analysed the data and interpreted the results. LP performed experiments. CB designed the Neapolis protocol and collected the samples. CVA, VT, LL, FMF, MEK and WM analysed the data. GC analysed the data and wrote the manuscript. LE designed the project, analysed the data, interpreted the results and wrote the manuscript.

## Conflict of interest

The authors declare that they have no conflict of interest.

## For more information

https://www.humanitas‐research.org/groups/leonardo‐elia/

https://www.unibs.it/ugov/person/154717

https://lion‐hearted.eu

## Supporting information



Appendix

Expanded View Figures PDF

Table EV1

Table EV2

Table EV3

Table EV4

Dataset EV1

Source Data for Expanded View and Appendix

Source Data for Figure 6

Source Data for Figure 7

## Data Availability

The data sets produced in this study are available in the following databases: https://www.ncbi.nlm.nih.gov/geo/query/acc.cgi?acc=GSE180017. The authors declare that all data supporting the findings of this study are available within the article and its supplementary information files.
