# Supplementary material for: rs41291957 controls miR‐143 and miR‐145 expression and impacts coronary artery disease risk
Source: EMBO Mol Med. 2021 Sep 22;13(10):e14060. doi: 10.15252/emmm.202114060 (PMC8495461; doi:10.15252/emmm.202114060)
Supplement: Supplementary file 1 — Appendix [file EMMM-13-e14060-s011.pdf]

APPENDIX

Table of contents

Appendix Fig S1 . . . . **2**

Appendix Fig S2. . . . . **3**

Appendix Fig S3. . . . . **4**

Appendix Fig S4. . . . . **5**

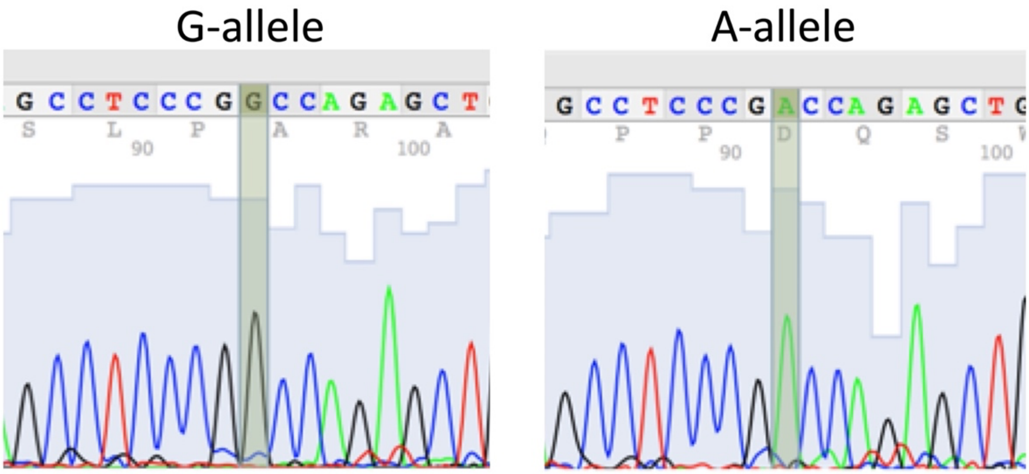

Appendix Fig S1. Generation of SNP-miR vectors.

Sanger sequence profiles of the miR-143/145 expression vectors utilized in the experiments shown in Fig 5.

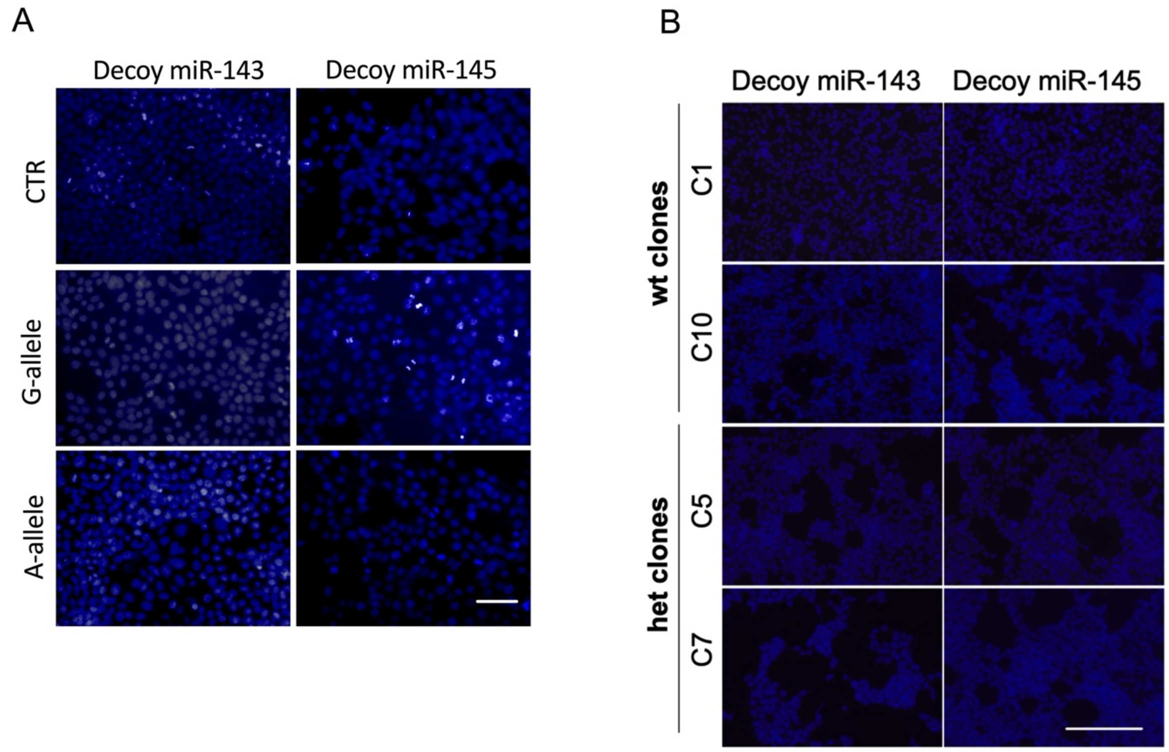

Appendix Fig S2. DAPI staining related to the images shown in Figures 4B (A) and 5C (B). Scale bars: 50µm.

A

gaaacaaagaaaagaagagaaaaaaggtcaaggtttggtcctgggtgctcaaatggcagg
*CCA*cagacaggaaacacagttgtgaggaattacaacagcctcccg**G**ccagagctggag
aggtggagcccaggtcccctctaacaccccttctcctggccaggttggagtcccgccacagg
ccaccagagcggagca**GCGCAGCGCCCTGTCT**

B

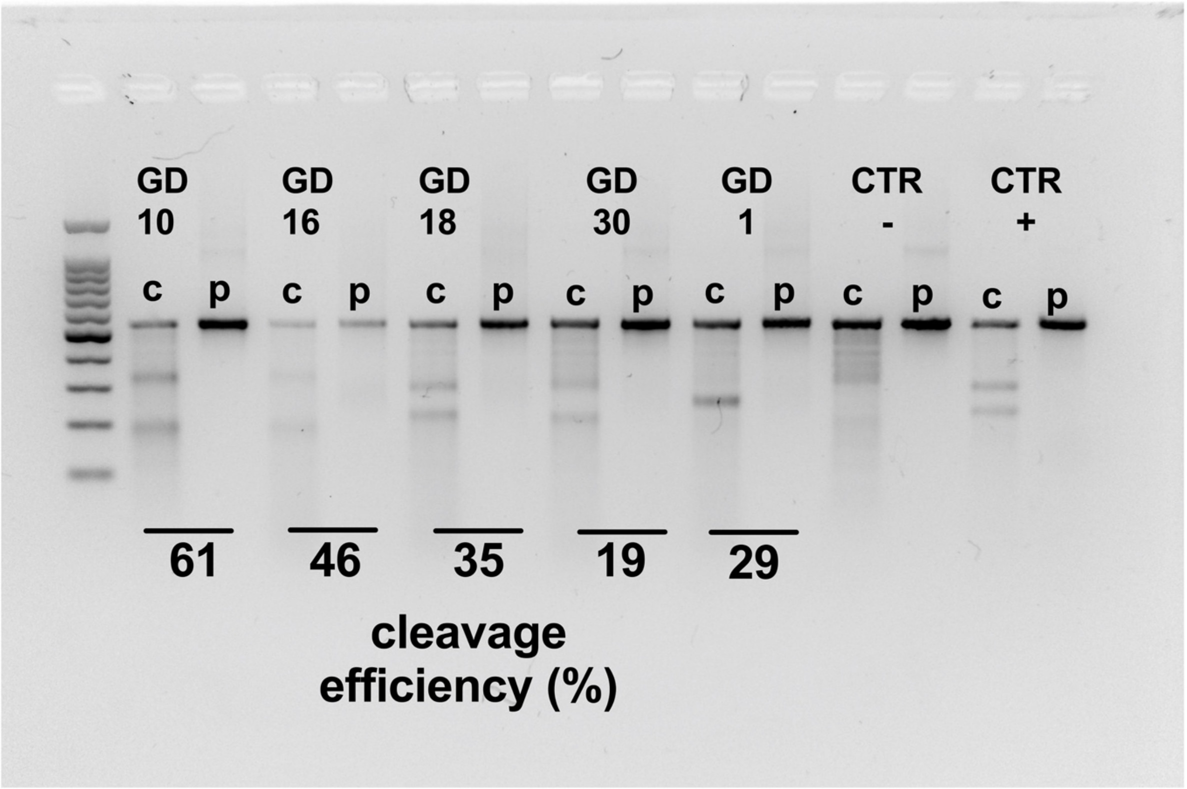

Appendix Fig S3. Generation of CRISPR/CAS9 clones.

(A) Wild-type sequence of the genomic locus including *rs41291957*. The genetic variation was introduced using an oligonucleotide for HR carrying the A-allele. The oligonucleotide sequence is underlined and includes an A instead of a G; the PAM sequence of the selected sgRNA is identified in italics but is on the opposite strand (*NGG*); the sequence of the mature miR-143 sequence is shown in bold. (B) Guide efficiency test.

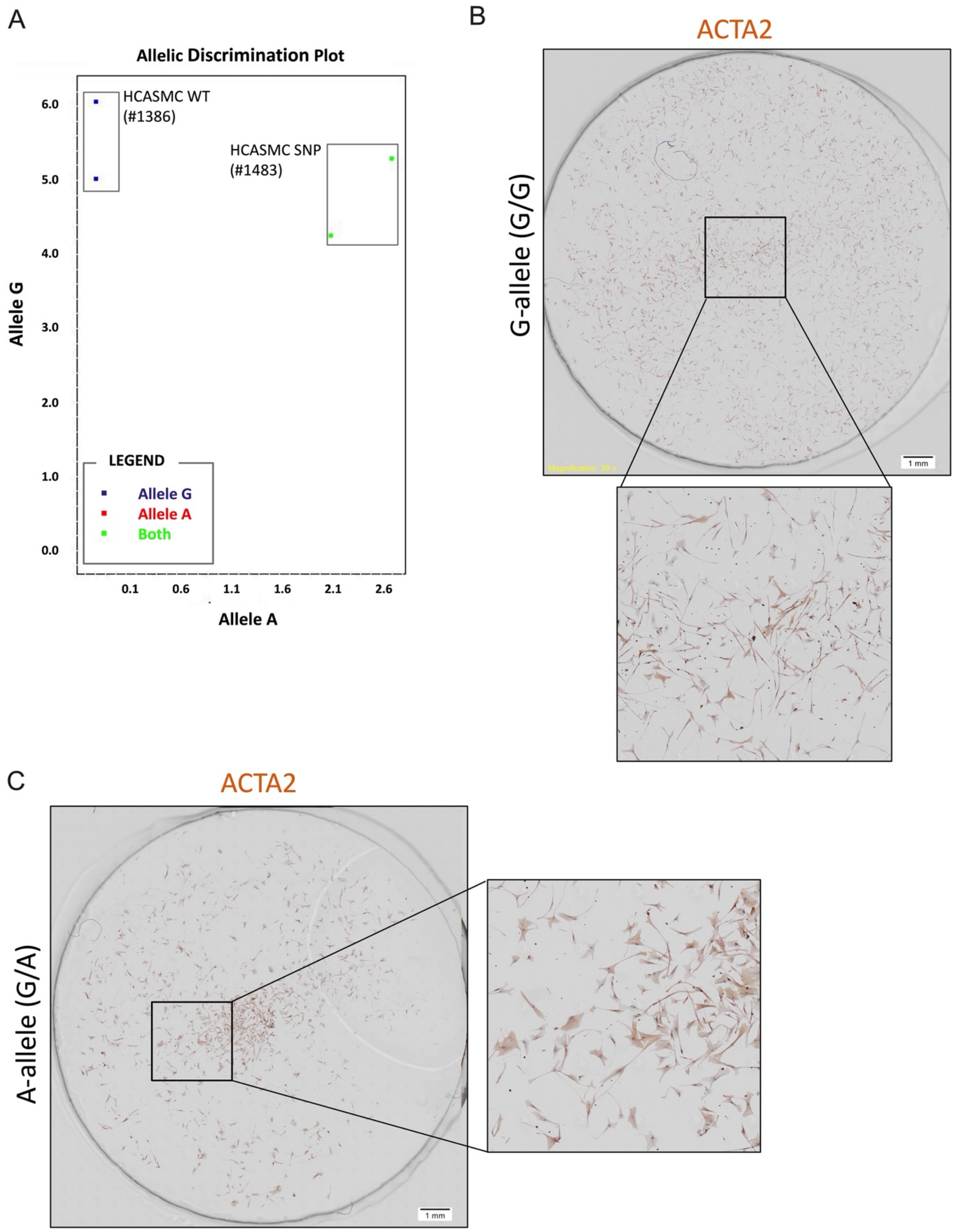

Appendix Fig S4. Allele discrimination analysis and ACTA2 staining of G- and A-allele HCASMCs.

(A) HCASMCs were genotyped also by qPCR using taqman probes. (B-C) ACTA2 staining of WT (B) and SNP (C) HCASMCs.
