# Supplementary material for: rs41291957 controls miR‐143 and miR‐145 expression and impacts coronary artery disease risk
Source: EMBO Mol Med. 2021 Sep 22;13(10):e14060. doi: 10.15252/emmm.202114060 (PMC8495461; doi:10.15252/emmm.202114060)
Supplement: Supplementary file 2 — Expanded View Figures PDF [file EMMM-13-e14060-s008.pdf]

Expanded View Figures

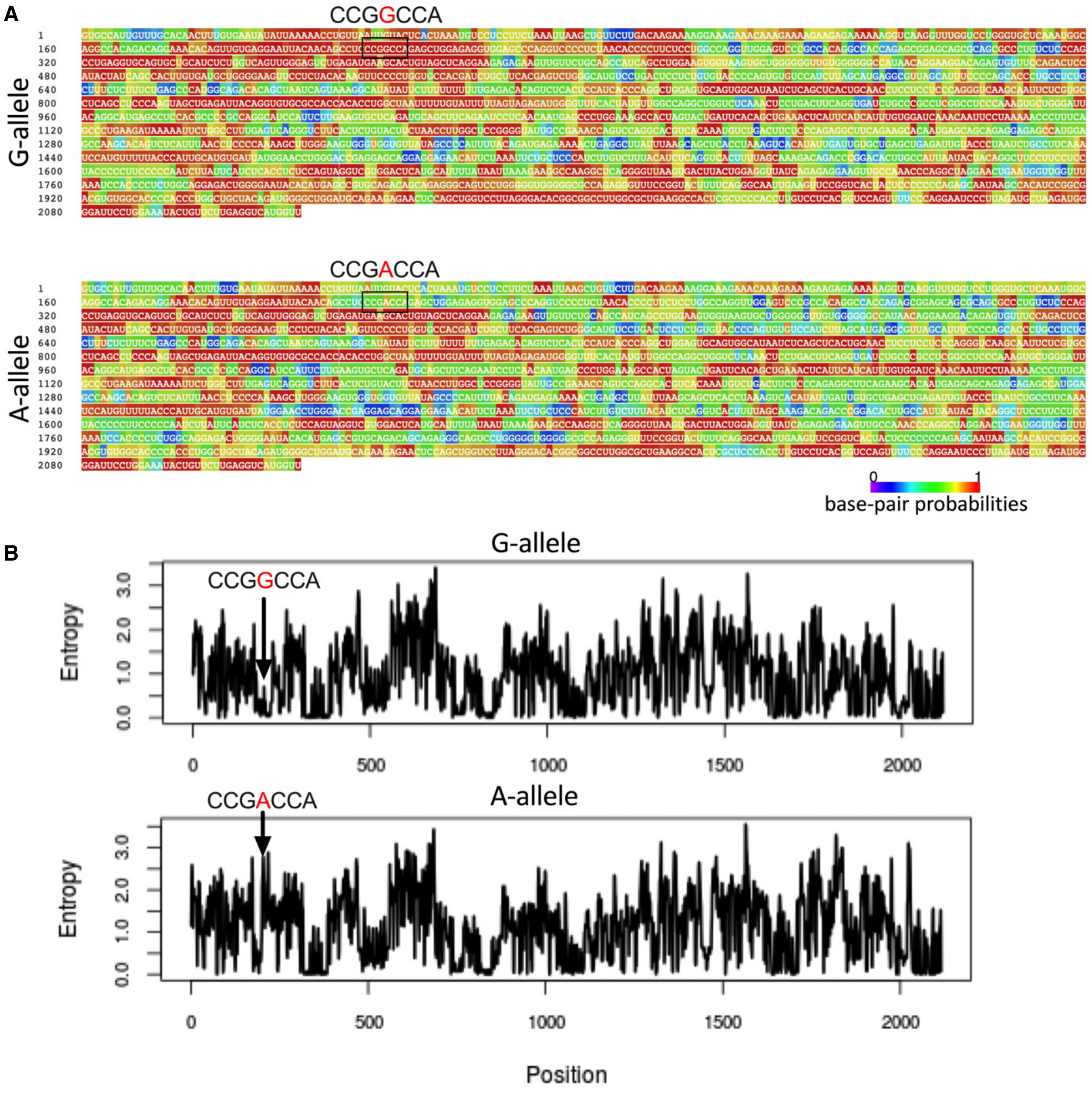

Figure EV1. Thermodynamic analysis of the miR-143/145 primary sequence.

A Base pair annealing probability using a centromeric prediction algorithm was carried out for the G- and A-allele sequences (the base in red indicates the variation).

B Entropy level profiles of the G- and A-allele transcripts.

© 2021 The Authors

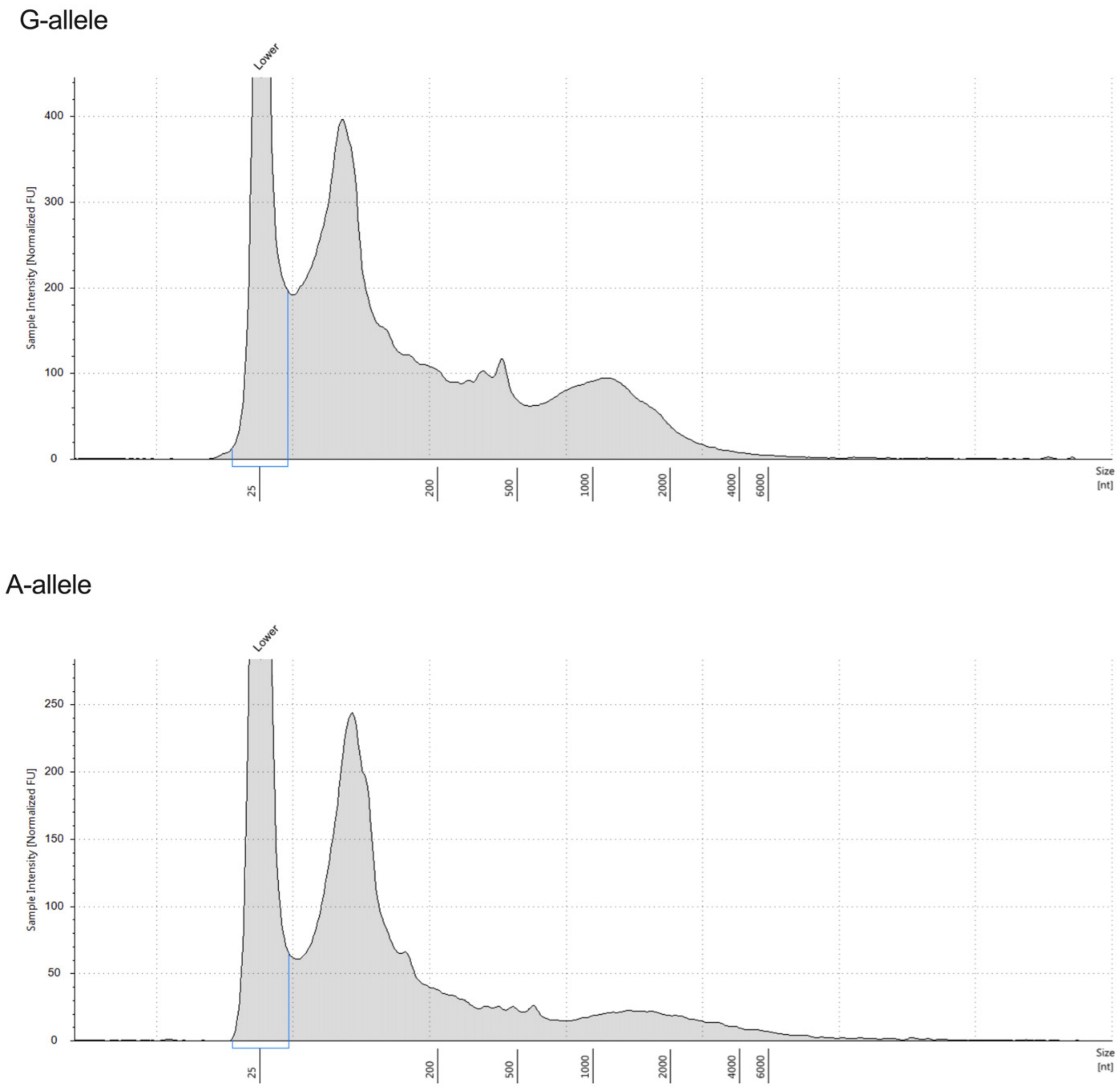

Figure EV2. TapeStation profiles of the G- and A-allele digested pri-miR-143/145.

 © 2021 The Authors

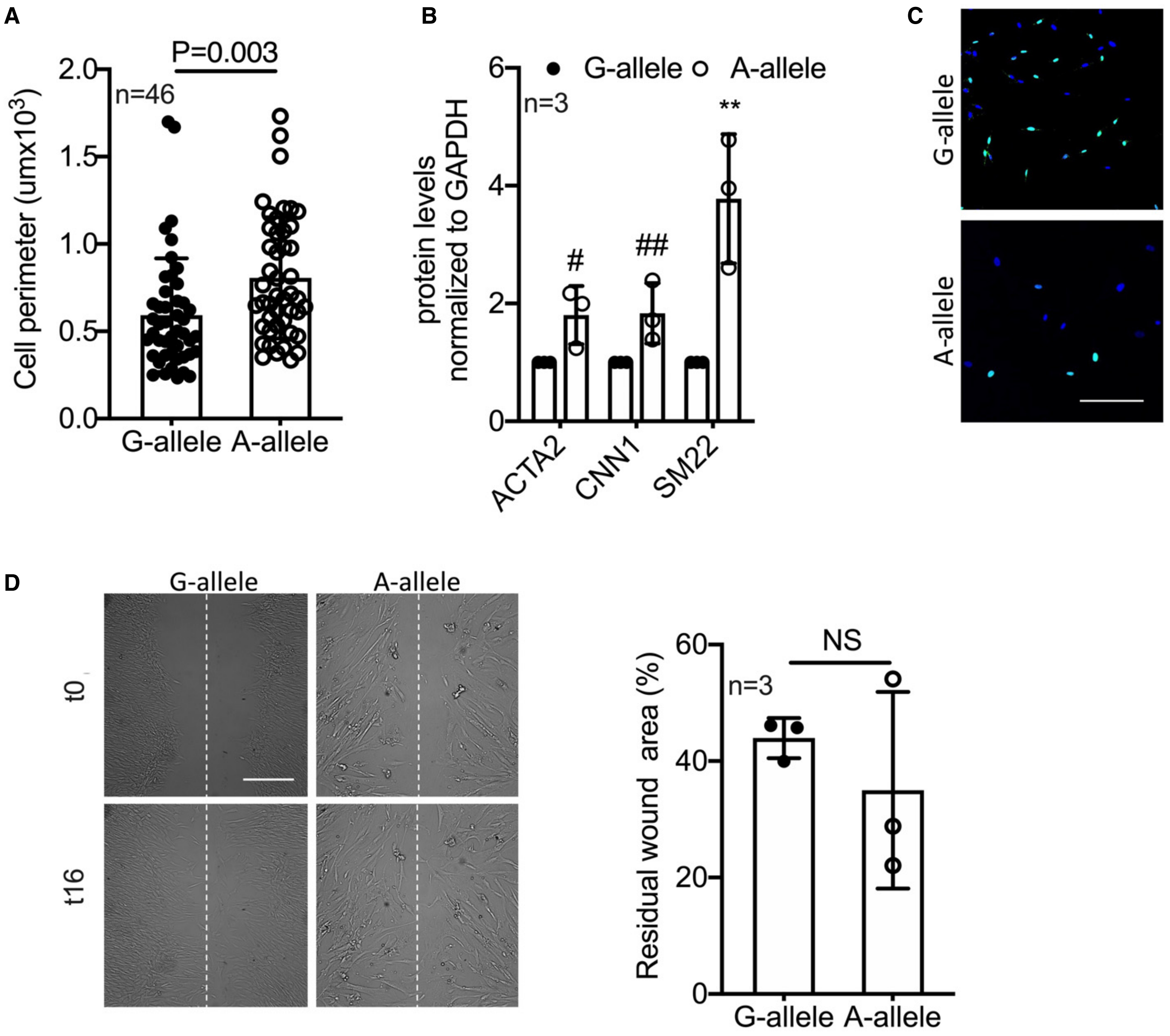

Figure EV3. HCASMC biological feature analysis.

A Quantification of the perimeter of HCASMCs carrying the G- and A-allele (n = 46).

B Quantification of Western blots in Fig 6G (n = 3).

C Representative picture of BrdU incorporating HCASMCs. Scale bar: 50 µm.

D Migration properties measured by scratch (n = 3). Scale bar 250 µm.

Data information: Data are shown as mean ± standard deviation (SD), and n indicates the number of biological replicates. To compare means, unpaired Student's t-test was used considering data from G-allele as control. For B: $^{\#}P$ = 0.0048, $^{\#\#}P$ = 0.0047, $^{**}P$ = 0.011. NS: not statistically significant.

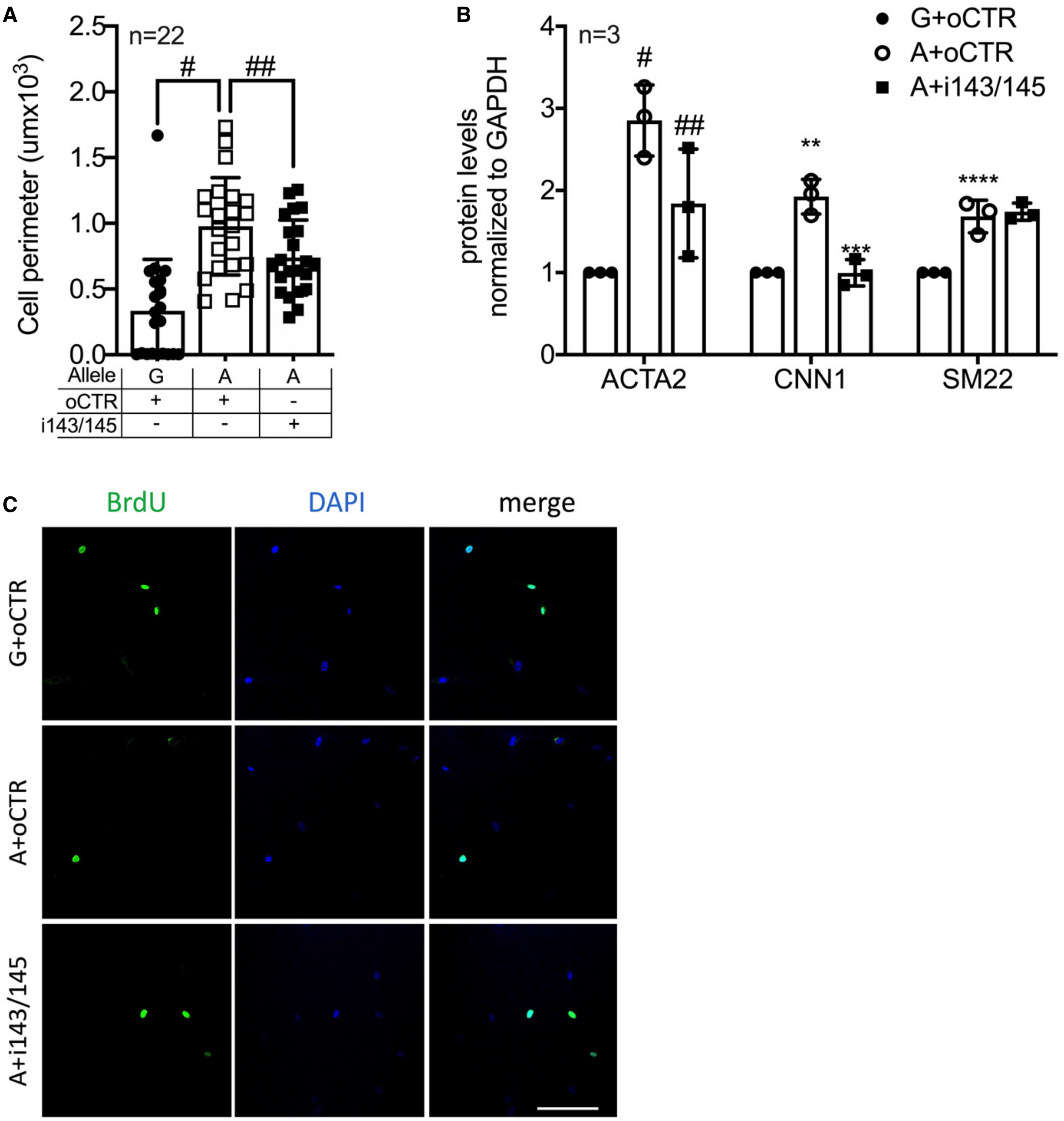

Figure EV4. miR-143 and miR-145 inhibition affects HCASMCs biological features.

A Quantification of size of HCASMCs carrying either the G- or A-allele transfected with a control oligonucleotide (oCTR) or after miR-143/145 inhibition (i143/145) (n = 22).

B Quantification of Western blots in Fig 7E (n = 3).

C Representative picture of BrdU incorporation. Scale bar: 50 μm.

Data information: Data are shown as mean ± standard deviation (SD), and n indicates the number of biological replicates. To compare means, one-way ANOVA with Tukey's multiple comparisons test was used. For A: #Adj P = 0.0001, ##Adj P = 0.0475; For B: #Adj P = 0.032, ##Adj P = 0.033, **Adj P = 0.03, ***Adj P = 0.025, ****Adj P = 0.048.

 © 2021 The Authors

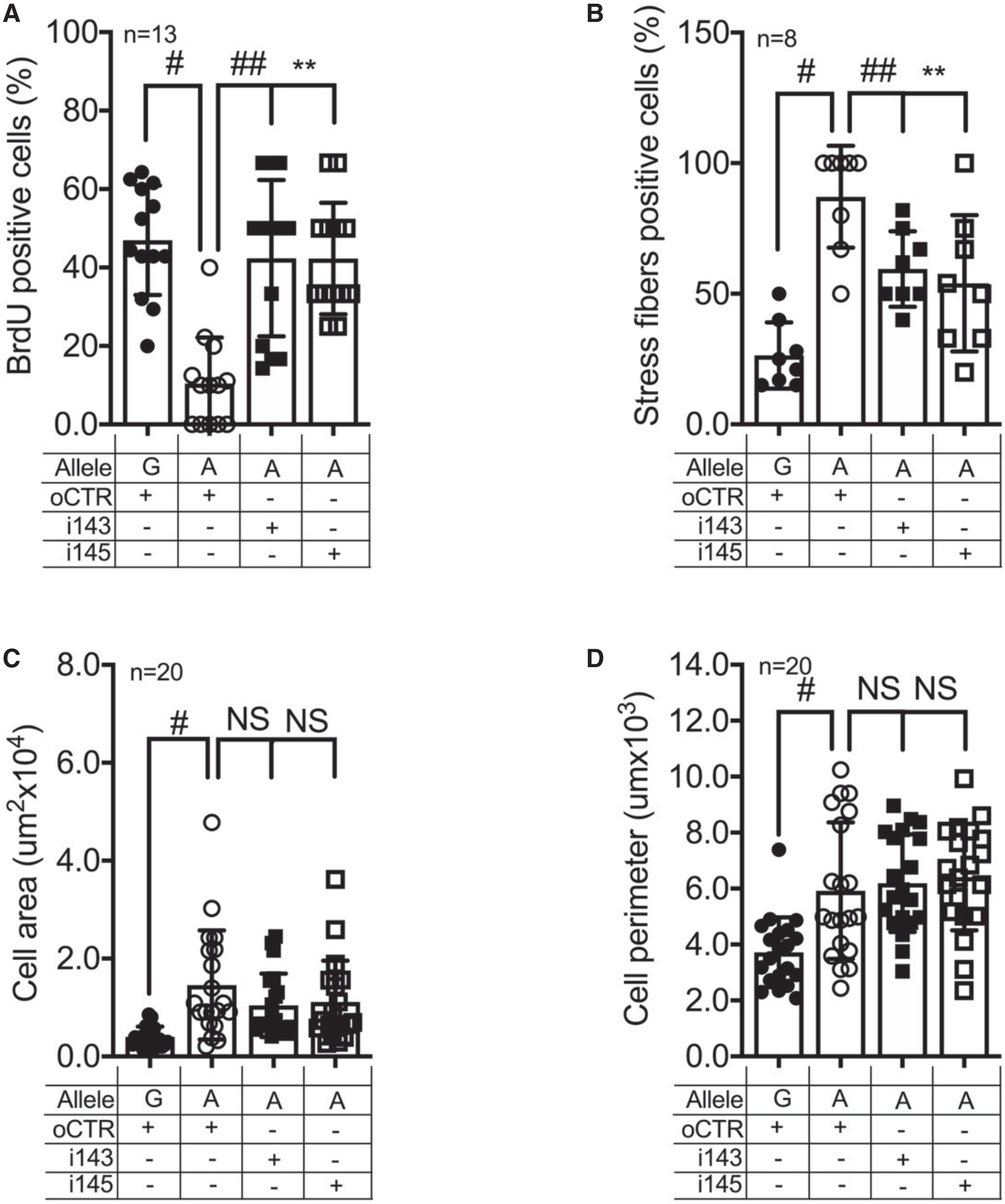

Figure EV5. miRNA-143 or 145 silencing partially rescues the effects of miR-SNP *rs41291957* on HCASMCs.

A Proliferation assay measured by BrdU incorporation in the rescue setting ($n = 13$).
B Quantification of actin signal fluorescence organized in stress fibres in the rescue setting ($n = 8$).
C Quantification of cellular area of HCASMCs in the rescue setting ($n = 20$).
D Quantification of cellular perimeter of HCASMCs in the rescue setting ($n = 20$).

Data information: Data are shown as mean ± standard deviation (SD), and *n* indicates the number of biological replicates. To compare means, one-way ANOVA with Tukey's multiple comparisons test was used. For A: $^{\#}$Adj $P = 0.0001$, $^{\#\#}$Adj $P = 0.0026$, **Adj $P = 0.0001$; For B: $^{\#}$Adj $P = 0.0001$, $^{\#\#}$Adj $P = 0.039$, **Adj $P = 0.011$; For C: $^{\#}P = 0.0015$; For D: $^{\#}P = 0.0026$. NS: not statistically significant.

© 2021 The Authors
