# Supplementary material for: rs41291957 controls miR‐143 and miR‐145 expression and impacts coronary artery disease risk
Source: EMBO Mol Med. 2021 Sep 22;13(10):e14060. doi: 10.15252/emmm.202114060 (PMC8495461; doi:10.15252/emmm.202114060)
Supplement: Supplementary file 3 — Table EV1 [file EMMM-13-e14060-s002.docx]

**Table EV1. CRISP/Cas9 guides and primers used in this study**

| **CRISP/Cas9 oligonucleotides** | | | |
| --- | --- | --- | --- |
| **ID** | **Forward** | **Reverse** |  |
| Guide 1 | CACCGACAACTGTGTTTCCTGTCTG | AAACCAGACAGGAAACACAGTTGTC |  |
| Guide 10 | CACCGAGGGGTGTTAGAGGGGACCT | AAACAGGTCCCCTCTAACACCCCTC |  |
| Guide 16 | CACCGAAGGGGTGTTAGAGGGGACC | AAACGGTCCCCTCTAACACCCCTTC |  |
| Guide 18 | CACCGCCTCTCCAGCTCTGGCCGGG | AAACCCCGGCCAGAGCTGGAGAGGC |  |
| Guide 30 | CACCGTCCACCTCTCCAGCTCTGGC | AAACGCCAGAGCTGGAGAGGTGGAC |  |
| HR oligo | TGCTCAAATGGCAGGCCACAGACAGGAAACACAGTTGTGAGGAATTACAACAGCCTCCCGACCAGAGCTGGAGAGGTGGAGCCCAGGTCCCCTCTAACACCCCTTCTCCTGGCCAGGTTG | |  |
| **RT-qPCR primers for gene expression** | | |  |
| \| **ID** \| **Forward** \| \| **Reverse** \| \| --- \| --- \| --- \| --- \| \| pri-miR-143/5 \| \| CAAAGACAGACCCGGACACT \| TGCATGAGTCCACGACCTAC \| \| U6 \| CGCTTCGGCAGCACATATAC \| \| TTCACGAATTTGCGTGTCAT \| \| hACTA2 \| CACCACTATGTACCCTGGAC \| \| TGATCTTCATGGTGCTGGGT \| \| hSM22 \| GGAAAGCATGTCATTGGCCT \| \| AACTGATGATCTGCCGAGGT \| \| hCNN1 \| AAGGTGAACGTGGGAGTGAA \| \| TCCGCCCTTCTCTTAGCTTC \| \| hCHP \| CTCCTTTGAGCTGTTTGCAG \| \| CACCACATGCTTGCCATCC \| | | |  |

| **Decoy sequences** |
| --- |
| \| **ID** \|  \| \| --- \| --- \| \| Decoy-miR-143 \| TCGAGTGAGCTACAGTGCTTCATCTCAAGAGAACTTAGAGAACTTTGAGCTACAGTGCTTCATCTCAT \| \| Decoy-miR-145 \| TCGAGTGAGGGATTCCTGGGAAAACTGGACAGAGAACTTAGAGAACTTTAGGGATTCCTGGGAAAACTGGACT \| |

| **RT-qPCR primers for cleavage analysis** |
| --- |
|  |
| \| **ID** \| **Forward** \| \| **Reverse** \| \| --- \| --- \| --- \| --- \| \| pri-miR-143/5 \| \| CCCTCTAACACCCCTTCTCC \| TCTCAGACTCCCAACTGACCA \| |
