# Supplementary material for: rs41291957 controls miR‐143 and miR‐145 expression and impacts coronary artery disease risk
Source: EMBO Mol Med. 2021 Sep 22;13(10):e14060. doi: 10.15252/emmm.202114060 (PMC8495461; doi:10.15252/emmm.202114060)
Supplement: Supplementary file 4 — Table EV2 [file EMMM-13-e14060-s001.docx]

| **Table EV2. Clinical and angiographic characteristics of Neapolis patients stratified for *rs41291957* polymorphisms** | | | | |
| --- | --- | --- | --- | --- |
|  | **Overall**  **(n=1726)** | ***rs41291957*** | |  |
|  |  | **GA+AA** | **GG** | **p Value** |
|  |  | **(n=327)** | **(n=1399)** |  |
| Clinical characteristics |  |  |  |  |
| Age, years | 65.6 ± 10.2 | 65.0 ± 9.9 | 65.7 ± 10.3 | 0.27 |
| Body mass index, Kg/m^2^ | 27.7 ± 4.3 | 27.9 ± 5.0 | 27.7 ± 4.1 | 0.41 |
| Men | 1332 (77.2) | 252 (77.1) | 1080 (77.2) | 0.94 |
| Family history | 417 (24.2) | 75 (22.9) | 342 (24.4) | 0.54 |
| Current smokers | 416 (24.1) | 71 (21.7) | 345 (24.7) | 0.36 |
| Dyslipidemia | 757 (43.8) | 152 (46.4) | 605 (43.2) | 0.42 |
| Hypertension | 1288 (74.6) | 231 (70.6) | 1007 (72.0) | 0.84 |
| Diabetes mellitus | 579 (33.5) | 130 (39.7) | 449 (32.1) | 0.01 |
| LVEF <30% | 62 (3.6) | 13 (4.0) | 49 (3.5) | 0.72 |
| CKD | 246 (14.25) | 57 (17.4) | 189 (13.5) | 0.07 |
| Prior MI | 662 (38.4) | 118 (36.1) | 544 (38.9) | 0.37 |
| Prior PCI | 545 (31.6) | 99 (30.3) | 446 (31.9) | 0.55 |
| Prior CABG | 213 (12.3) | 40 (12.2) | 173 (12.4) | 0.95 |
| Angiographic characteristics |  |  |  |  |
| Multivessel disease |  |  |  | 0.68 |
| 1-vessel | 460 (26.7) | 81 (24.8) | 379 (27.1) | 0.39 |
| 2-vessel | 605 (35.1) | 113 (34.6) | 492 (35.2) | 0.83 |
| 3-vessel | 594 (34.4) | 119 (36.4) | 475 (34.0) | 0.40 |
| Stent length, mm | 27.6 ± 12.1 | 27.6 ± 12.1 | 27.7 ± 12.2 | 0.93 |
| Complex lesion (B2/C) | 1071 (62.0) | 201 (61.4) | 870 (62.1) | 0.93 |
| Chronic total occlusion | 178 (10.3) | 24 (7.3) | 154 (11.0) | 0.049 |
| Bifurcation lesion | 291 (16.9) | 51 (15.6) | 240 (17.2) | 0.52 |
| Calcified lesion | 381 (22.1) | 73 (22.3) | 308 (22.0) | 0.90 |
| Thrombotic lesion | 95 (5.5) | 14 (4.3) | 81 (5.8) | 0.29 |

Values are n (%) or mean±SD.

LVEF=left ventricular ejection fraction, CKD=chronic kidney disease, MI=myocardial infarction, PCI=percutaneous coronary intervention, CABG=coronary artery bypass grafting.
