# Supplementary material for: rs41291957 controls miR‐143 and miR‐145 expression and impacts coronary artery disease risk
Source: EMBO Mol Med. 2021 Sep 22;13(10):e14060. doi: 10.15252/emmm.202114060 (PMC8495461; doi:10.15252/emmm.202114060)
Supplement: Supplementary file 5 — Table EV3 [file EMMM-13-e14060-s005.docx]

| **Table EV3. Clinical and angiographic characteristics of LURIC patients stratified for *rs41291957* polymorphisms** | | | | |
| --- | --- | --- | --- | --- |
|  | **Overall**  **(n=2601)** | ***rs41291957*** | |  |
|  |  | **GA+AA** | **GG** | **p Value** |
|  |  | **(n=532)** | **(n=2069)** |  |
| ***Clinical characteristics*** |  |  |  |  |
| Age, years | 62.6±10.6 | 62.9±10.5 | 62.6±10.6 | 0.549 |
| Body mass index, Kg/m^2^ | 27.4±4.1 | 27.5±4.13 | 27.4±4.07 | 0.462 |
| Men | 1834 (70.5) | 364 (68.4) | 1470 (71.0) | 0.236 |
| Family history (of MI) | 928 (35.7) | 166 (31.2) | 762 (36.8) | 0.016 |
| Current or ex-smokers | 1668 (64.1) | 330 (62.0) | 1338 (64.7) | 0.258 |
| Dyslipidemia | 1780 (68.4) | 343 (64.5) | 1437 (69.5) | 0.028 |
| Hypertension | 1887 (72.5) | 392 (73.7) | 1495 (72.3) | 0.511 |
| Diabetes mellitus | 1033 (39.7) | 214 (40.2) | 819 (39.6) | 0.787 |
| LVEF <30% | NA | - | - | - |
| CKD | 355 (13.6) | 84 (15.8) | 271 (13.1) | 0.107 |
| Prior MI | 1091 (41.9) | 202 (38.0) | 889 (43.0) | 0.037 |
| Prior PCI | 717 (27.6) | 131 (24.9) | 586 (28.5) | 0.092 |
| Prior CABG | 331 (12.7) | 68 (12.8) | 263 (12.7) | 0.965 |
| Angiographic characteristics |  |  |  |  |
| Multivessel disease |  |  |  |  |
| 1-vessel | 495 (19.0) | 94 (17.7) | 401 (19.4) | 0.37 |
| 2-vessel | 491 (18.9) | 100 (18.8) | 391 (18.9) | 0.958 |
| 3-vessel | 788 (30.3) | 155 (29.1) | 633 (30.6) | 0.514 |
| Stent length, mm | NA | - | - | - |
| Complex lesion (B2/C) | NA | - | - | - |
| Chronic total occlusion | NA | - | - | - |
| Bifurcation lesion | NA | - | - | - |
| Calcified lesion | NA | - | - | - |
| Thrombotic lesion | NA | - | - | - |

Values are n (%) or mean±SD.

LVEF=left ventricular ejection fraction, CKD=chronic kidney disease, MI=myocardial infarction, PCI=percutaneous coronary intervention, CABG=coronary artery bypass grafting. NA=Data not available
