# Supplementary material for: rs41291957 controls miR‐143 and miR‐145 expression and impacts coronary artery disease risk
Source: EMBO Mol Med. 2021 Sep 22;13(10):e14060. doi: 10.15252/emmm.202114060 (PMC8495461; doi:10.15252/emmm.202114060)
Supplement: Supplementary file 6 — Table EV4 [file EMMM-13-e14060-s006.docx]

| **Table EV4. Clinical and angiographic characteristics of Neapolis patients stratified for the presence of chronic total occlusion** | | | |
| --- | --- | --- | --- |
|  | **Without CTO**  **(n = 1548)** | **With CTO**  **(n = 178)** | **p Value** |
| Male | 1197 (77.3) | 135 (75.8) | 0.64 |
| Age, yrs | 65.5 ± 10.36 | 66.2 ± 9.16 | 0.34 |
| Family History | 380 (24.54) | 37 (20.78) | 0.25 |
| Body mass index, kg/m^2^ | 27.73 ± 4.36 | 27.73 ± 4.01 | 0.98 |
| Current smokers | 375 (24.22) | 41 (23.03) | 0.86 |
| Dyslipidemia | 680 (43.9) | 77 (43.2) | 0.87 |
| Hypertension | 1246 (79.16) | 142 (9.1) | 0.08 |
| LVEF <30% | 52 (3.35) | 10 (5.61) | 0.19 |
| Diabetes mellitus | 514 (33.2) | 65 (36.5) | 0.63 |
| CKD | 211 (13.6) | 35 (19.6) | 0.32 |
| Prior MI | 586 (37.85) | 76 (42.69) | 0.21 |
| Prior PCI | 501 (32.36) | 44 (24.71) | 0.03 |
| Prior CABG | 190 (12.27) | 23 (12.92) | 0.8 |
| Multivessel disease |  |  | 0.09 |
| 1-vessel | 404 (26.09) | 56 (31.46) | 0.15 |
| 2-vessels | 539 (34.81) | 66 (37.07) | 0.56 |
| 3-vessels | 541 (34.94) | 53 (29.77) | 0.16 |
| Stent length, mm | 26.84 ± 11.59 | 35.11 ± 14.42 | <0.0001 |
| Complex lesion (B2/C) | 897 (57.94) | 174 (97.7) | <0.0001 |
| Bifurcation lesion | 274 (17.70) | 17 (9.55) | 0.004 |
| Calcified lesion | 398 (25.5) | 57 (32) | 0.06 |
| Thrombotic lesion | 69 (4.4) | 26 (14.5) | <0.0001 |

Values are n (%) or mean ± SD.

CABG=coronary artery bypass surgery; CKD=estimated glomerular filtration rate; CTO=chronic total occlusion; LVEF=left ventricular ejection fraction; MI=myocardial infarction; PCI=percutaneous coronary intervention.
