# Supplementary figures and images for: rs41291957 controls miR‐143 and miR‐145 expression and impacts coronary artery disease risk
Source: EMBO Mol Med. 2021 Sep 22;13(10):e14060. doi: 10.15252/emmm.202114060 (PMC8495461; doi:10.15252/emmm.202114060)

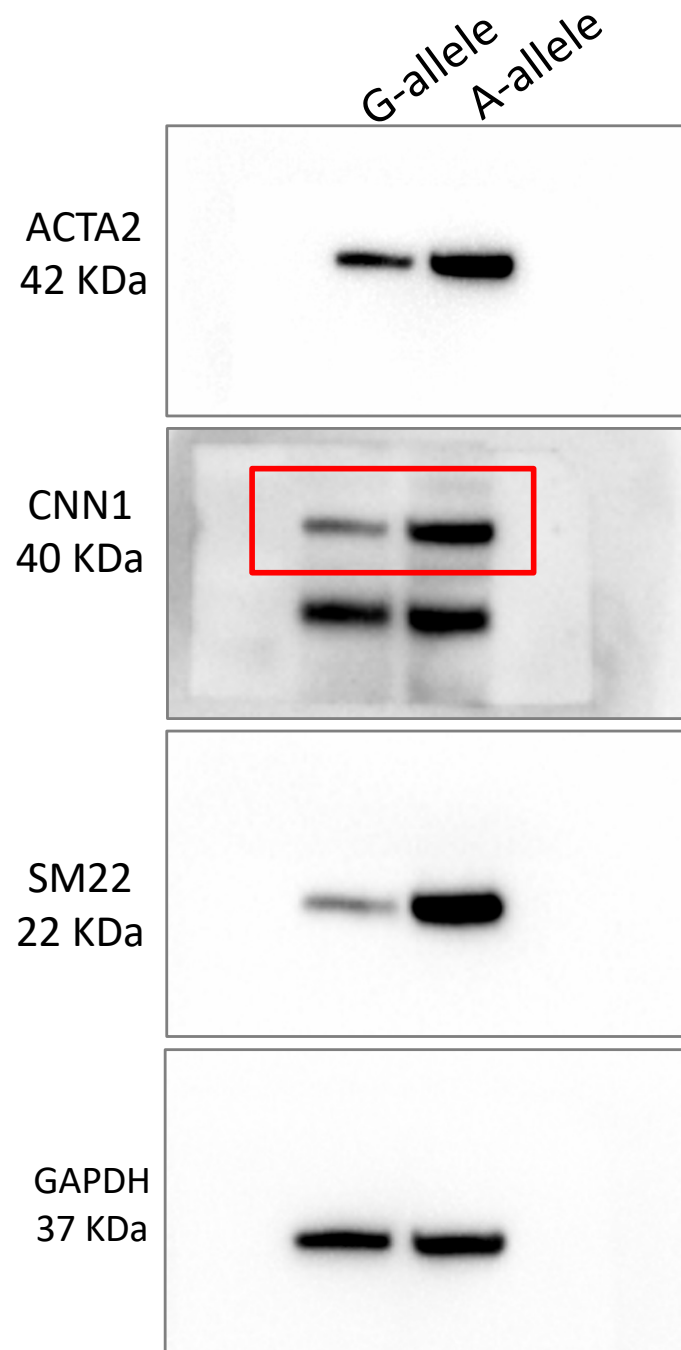

Full unedited blot for Figure 6G  
Acquired with ChemiDoc (BioRad)

Supplement: Supplementary file 9 — Source Data for Figure 6 [file EMMM-13-e14060-s009.pdf]

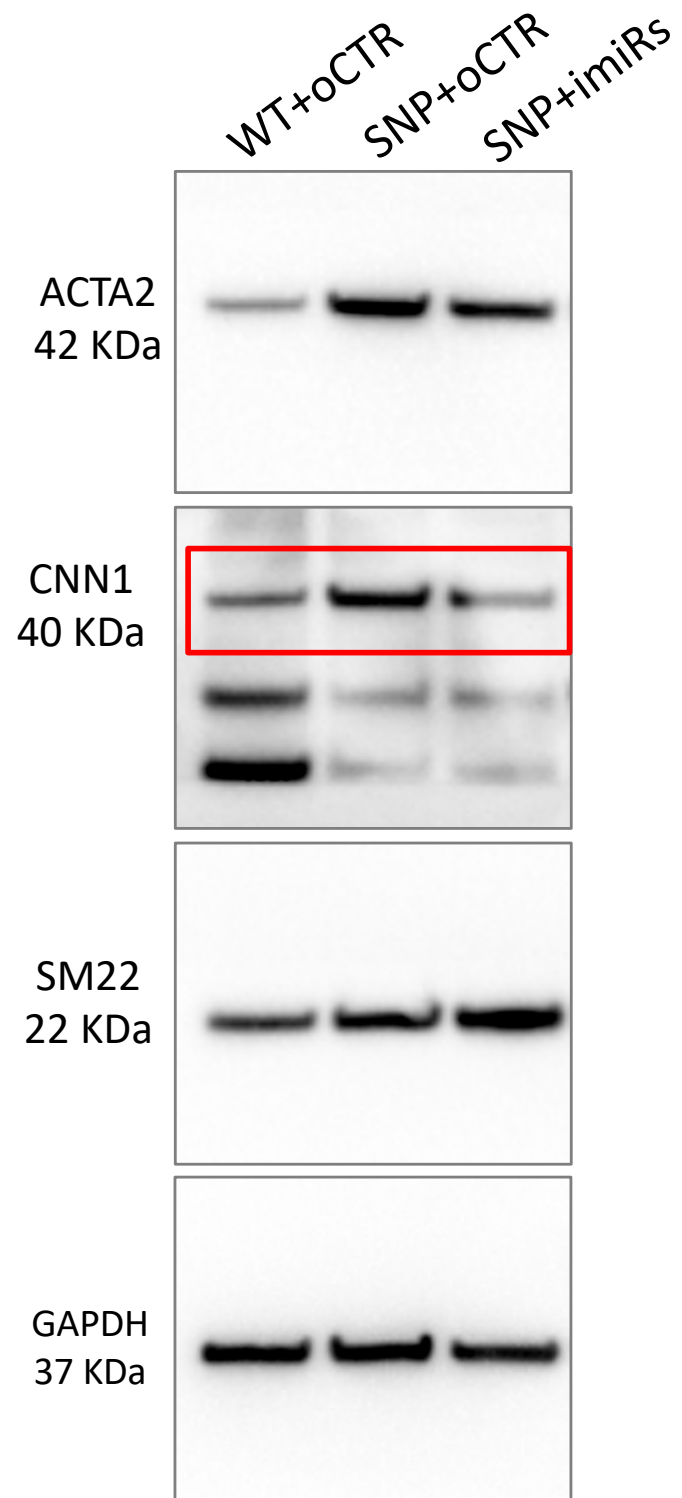

Full unedited blot for Figure 7E  
Acquired with ChemiDoc (BioRad)

Supplement: Supplementary file 10 — Source Data for Figure 7 [file EMMM-13-e14060-s003.pdf]
