# Supplementary material for: rs41291957 controls miR‐143 and miR‐145 expression and impacts coronary artery disease risk
Source: EMBO Mol Med. 2021 Sep 22;13(10):e14060. doi: 10.15252/emmm.202114060 (PMC8495461; doi:10.15252/emmm.202114060)
Supplement: Supplementary file 11 [file EMMM-13-e14060-s007.pdf]

EMBO Molecular Medicine

rs41291957 variant controls miR-143 and miR-145 expression and impacts Coronary Artery Disease risk

Leonardo Elia, Ignacio Hall, Climent Montserrat, Laura Papa, Vinicius Tragante, Floriana Farina, Markus Kleber, Winfried März, Carlo Biguori, Gianluigi Condorelli, Chiara Viviani, and Luca Lambroia
DOI: 10.15252/emmm.202114060

Corresponding author: Leonardo Elia (leonardo.elia@unibs.it)

Review Timeline:

Submission Date:	3rd Feb 21
Editorial Decision:	1st Mar 21
Revision Received:	9th Aug 21
Editorial Decision:	18th Aug 21
Revision Received:	24th Aug 21
Accepted:	24th Aug 21

Editor: Zeljko Durdevic

Transaction Report:

(Note: With the exception of the correction of typographical or spelling errors that could be a source of ambiguity, letters and reports are not edited. Depending on transfer agreements, referee reports obtained elsewhere may or may not be included in this compilation. Referee reports are anonymous unless the Referee chooses to sign their reports.)

1st Editorial Decision 1st Mar 2021

1st Mar 2021

Dear Prof. Elia,

Thank you for the submission of your manuscript to EMBO Molecular Medicine. We have now received feedback from the three reviewers who agreed to evaluate your manuscript. As you will see from the reports below, the referees acknowledge the interest of the study but also raise serious concerns that should be addressed in a major revision.

Addressing the reviewers' concerns in full will be necessary for further considering the manuscript in our journal, and acceptance of the manuscript will entail a second round of review. EMBO Molecular Medicine encourages a single round of revision only and therefore, acceptance or rejection of the manuscript will depend on the completeness of your responses included in the next, final version of the manuscript. For this reason, and to save you from any frustrations in the end, I would strongly advise against returning an incomplete revision.

We realize that the current situation is exceptional on the account of the COVID-19/SARS-CoV-2 pandemic. Therefore, please let us know if you need more than three months to revise the manuscript.

I look forward to receiving your revised manuscript.

Yours sincerely,

Zeljko Durdevic
Editor
EMBO Molecular Medicine

***** Reviewer's comments *****

Referee #1 (Remarks for Author):

Hall et al. rs41291957 variant controls miR-143 and miR-145 expression and impacts Coronary Artery Disease risk

This manuscript by a group of very strong scientists studies SNP rs41291957 positioned -91 bp upstream of the miR-143/145 gene locus. Modelling supports the contention that an A-allele could facilitate processing of the primary miRNA. This was supported by careful control of transiently transfected plasmids and by CRISPR editing of the endogenous locus in HEK293 cells. The findings were followed up in two commercially available primary smooth muscle cell lines, one carrying the

reference (G/G) and one the mutant (G/A) rs41291957 allele, resulting in higher miR-143/145 expression and more differentiation, which could be blocked by antisense oligonucleotide interventions. In patient cohorts, presence of the rs41291957 allele correlated with a reduction in coronary artery disease events.

Major

1. In Figure 2, the individual sample size is somewhat low and significance depends on possible outliers. Increase the sample size would generate more statistical power. Also label the figures so that n number for each condition are immediately visible.
2. Figure 3, same comment as for Figure 2 and why was chosen for a Holm-Sidak's multiple comparison test instead of a Tukey's test where you compute confidence intervals for every comparison?
3. Figure 4: The difference in robustness of data becomes more clear when comparing the CRISPR edited clones from Fig 4 with the more variable effects seen with transient transfections in Figs 2,3.
4. Figure 4: how do the authors explain/interpret the expression differences between clones c1 and c10 only for miR-145?
5. Figure 5: the two different bars in panel B refer to different clones or technical replicates?
6. A major strength of the study involves the two separate and robust clinical cohorts (>1000 pts). Within these cohorts, significant differences in prevalence did exist (e.g. Neapolis: diabetes, CKD) between SNP carriers that are not easily explained by a smooth muscle phenotype. These should be amply discussed.
7. The authors should discuss more extensively their own previous findings in miR-143/145 knockout/overexxpressing mice as to avoid inclusion of an animal model in the present manuscript.

Minor
1. Consider rephrasing second sentence of Abstract: "... has been established not only for gene products but also for microRNAs (miRNAs)". Gene products probably refers to protein coding genes and microRNAs refers to microRNA genes or genes encoding microRNAs.
2. Rephrase sentence on page 14: "Secondly, the retrospective nature the clinical studies..."

Referee #2 (Comments on Novelty/Model System for Author):

the paper is technically very nicely executed but to me it lacks novelty (just another snp regulating a miRNA) and medical impact (there is no "medicine" in the paper)

Referee #2 (Remarks for Author):

In this manuscript, Hall and colleagues add new interesting insights to the role of single nucleotide polymorphisms (SNPs) to coronary artery disease. The authors focus here on rs41291957 which upregulates miR-143 and miR-145, triggering the phenotypic switch of vascular smooth cells towards a more contractile phenotype. Furthermore, the authors propose a novel link between the expression of rs41291957 and a protective status in human CAD. Although other SNPs as rs353292 and rs4705343 have been shown to modulate the miRNA cluster miR143/145, this work in itself is novel.

While the manuscript of Hall et al. is carefully executed and technically sound, however I recommend

the authors to address the following points.

Minor comments

1. Figure 3B: The authors should try improving the quality of the pictures. Maybe with a higher magnification. Also, DAPI should be included as it is done in other figures for quantification.

2. Figure 4C: The authors should include DAPI staining pictures and bar scale information.

3. Figure 5: The effects in SMC differentiation are only moderate. The authors may consider including further differentiation markers or longer time points to enhance the effects.

4. Figure 5 H: The western blots should be n=3 and quantified. The authors should consider including further contractile markers (e.g. calponin, caldesmon, sm-actin).

5. Figure 6.F. the western blot would need to be n=3 and be quantified for normalization to GAPDH.

6. Please correct "Collagene" for collagen: Fig5L and Fig6H

Referee #3 (Comments on Novelty/Model System for Author):

The findings are novel and clearly of interest to the field. The statistical analysis might benefit from an additional statistical review.

Referee #3 (Remarks for Author):

In this work Hall and colleagues elegantly analysed the effect of a single nucleotide polymorphism (SNP) identified in the miR-143/145 cluster gene. Date reported in their work indicated that the SNP with a minor allele frequency of at least 10% was protective in coronary artery disease (CAD) patients. The miR-143/145 cluster is predominantly expressed in vascular smooth muscle cells and regulates their phenotypic switch, crucial in several cardiovascular diseases, such as atherosclerosis, CAD, and arterial hypertension.
The work is very well presented, and experiments have been properly designed and discussed. The rationale is clear, and data here reported support the existence of a SNP able to confer protection in certain patients affected by artery disease. This reviewer has some concerns that authors should address to improve their work:

1. Why did the authors, among all miRNAs and miRNA clusters that are known to play key roles in CAD decide for the miR-143/145 cluster? This point should be better outlined and discussed in the introduction. Here, the authors should include other relevant miRNAs, for which SNP have been identified to underline the rationale behind the selection of miR-143/145 cluster. Indeed, athero-prone miRNAs are known to contain SNPs and not all have been studied so far.
2. The authors identified the rs41291957 SNP. The ID correspond to two different mutations reported, namely 148808390 G>A and 149428927 G>A. Which one are authors are referring to? What is the frequency and difference (if any) between the two?
3. Authors indicated that the G>A mutation generates a potential less complex secondary structure of the miRNA precursor, leading to an increased accessibility for the miRNA maturation

enzymes. However, the prediction obtained is merely computational and based on a centromeric prediction. Authors should explain the parameters used in more detail. First, did authors considered the G:U pairs? What is the rationale of a centroid prediction instead of a minimum free energy secondary structure?
4. On a related note and to support the hypothesis of a different accessibility of the enzyme at the secondary structure, the authors should perform an enzymatic cleavage of single strand RNA, followed by a sequencing or similar of the RNA:RNA secondary structures. The loops should be protected from the cleavage and show the differences in the secondary structures. Ideally, the authors should perform an immunoprecipitation to see if any difference occurs in the binding affinity in G>A group. This is fundamental to support the RNA secondary structure accessibility and prediction.
5. Statistical analysis should be included and better elaborated in the material and methods as paragraph, and not only in the figure legends.
6. The data reported in Figure 4B indicated that C10 clone significantly reduced miR-145 but not miR-143 levels. How do author explain this?
7. The miR-143 and miR-145 can play different roles. Which is the effect of the SNP on their individual function?
8. The authors focused on GA in heterozygosis. However, AA homozygosis have been reported in patients. The authors should explain the reason for focusing on heterozygosis and introduce the effect of AA homozygosis. This could be particularly important with regards to future potential therapies aiming the analysis of the SNP in other diseases and cell types. Related to this, the authors describe the AA + GA data from patients but they did not investigate it in vitro. These data would be very instructive concerning the existence of patients carrying the AA homozygosis. Moreover, how do authors explain the higher rate of diabetes? This should be discussed or ideally studied, at least in vitro.
9. There is a difference between C5 and C7 clones on miR-143 and miR-145 expression, which is opposite. How can authors explain it? How can they have the final effect on GFP expression? is there any target saturation? Do the miRNAs show a competitive role in terms of co-factor accessibility?
10. What is the final conclusion that the authors would draw from the existence of the SNP? Is the frequency of the SNP the same in all different populations studied?

Minor concern:
1. Page 6, line 3: ensemble should be changed with ensembl
2. The legend of figure 6A is missing.

Referee #1

Remarks for Author

Hall et al. rs41291957 variant controls miR-143 and miR-145 expression and impacts Coronary Artery Disease risk. This manuscript by a group of very strong scientists studies SNP rs41291957 positioned -91 bp upstream of the miR-143/145 gene locus. Modelling supports the contention that an A-allele could facilitate processing of the primary miRNA. This was supported by careful control of transiently transfected plasmids and by CRISPR editing of the endogenous locus in HEK293 cells. The findings were followed up in two commercially available primary smooth muscle cell lines, one carrying the reference (G/G) and one the mutant (G/A) rs41291957 allele, resulting in higher miR-143/145 expression and more differentiation, which could be blocked by antisense oligonucleotide interventions. In patient cohorts, presence of the rs41291957 allele correlated with a reduction in coronary artery disease events.

We thank the referee for the positive comment on our group and work.

Major

1. In Figure 2, the individual sample size is somewhat low and significance depends on possible outliers. Increase the sample size would generate more statistical power. Also label the figures so that n number for each condition are immediately visible.

We thank the referee for his/her comment. However, it is not very clear to us the meaning of the sentence "the individual sample size is somewhat low and significance depends on possible outliers". All the data were derived from multiple biological replicates, made in different moments with different lots of cells, in order to obtain strong and reproducible results. Specifically, panels A and C include respectively n=10 and n=7, and although a certain variability was observed, we first executed a statistical test to identify possible outliers (ROUT test), with none being identified; then, we ran specific significance unpaired t-test, finding the clear statistical difference as indicated. Similarly, we ran the same statistical approach also for Figure 2B (Now Figure 3B), in which we had 3 independent experimental replicates. Probably, the referee comment is referring to the latest cited panel, for which we have now increased the number of tested samples (to n=5). We have also labelled the figures in order to show directly the n number. We have also better described in the method section all the statistical analyses performed.

2. Figure 3, same comment as for Figure 2 and why was chosen for a Holm-Sidak's multiple comparison test instead of a Tukey's test where you compute confidence intervals for every comparison?

As explained above, our experiments were performed in order to generate reproducible data, which were derived from independent biological replicates of at least three different experiments, as any statistical test requires. However, following the referee's request, we have now increased the number of replicates also for this specific experiment (to n=8; New Figure 4C)). On the other hand, we used Holm-Sidak's multiple comparison test because it is a more powerful method of computation compared to Tukey's test. However, following this specific request of the referee, we have now run Tukey's comparison test and new Adj P values have been calculated.

3. Figure 4: The difference in robustness of data becomes more clear when comparing

the CRISPR edited clones from Fig 4 with the more variable effects seen with transient transfections in Figs 2,3.

As the referee has noted here, the variability is lower, and clearly this depends on the fact that we worked with specific cellular clones, while in the above-mentioned experiments cells were transfected every time at different moments. This behavior is nothing unusual, and again the statistical test we utilized included always the evaluation of putative outliers. However, also for these experiments we have now increased the number of analyzed samples for Figure 5C (to n=6).

4. Figure 4: how do the authors explain/interpret the expression differences between clones c1 and c10 only for miR-145?

We thank the referee for the observation. We have thought about this difference, trying to come up with a reasonable answer. However, cells are genetically modified, and although the CRISPR system that we employed used two different gRNA guides in order to reduce the possibility of random mutation, we cannot exclude that something happened in the control cells (transfected with vectors with no gRNA). Therefore, the difference might depend on the modulation of another regulatory mechanism that we cannot identify. Nonetheless, the referee should take into consideration that the two WT clones had miR-145 expression levels that were always lower compared to the SNP ones, a fact strongly support our conclusion. Statistical analysis was performed considering all raw data derived from the qPCR experiments; for the DDct calculation, we selected as 1 only one sample (the C1 clone), and this generated a difference between the two WT clones. Overall, we are only able to speculate on the reduced expression of miR-145 in the C10 clone, but the important message here is that, independently from this difference, the SNP clones always have a higher level of expression of both miRNAs compared to the controls. Furthermore, during clone selection, we identified different WT clones, and then chose C1 and C10 based on their similarity to the original cells for phenotypic characteristics. For the referee's evaluation, we show here the miR-143 and -145 levels in a WT clone that was not utilized for the decoy experiments (Figure 1 for Referee1). As the referee can observe, the WT clone G1, despite some variability, shows a small increase in miR-145 compared to C1.

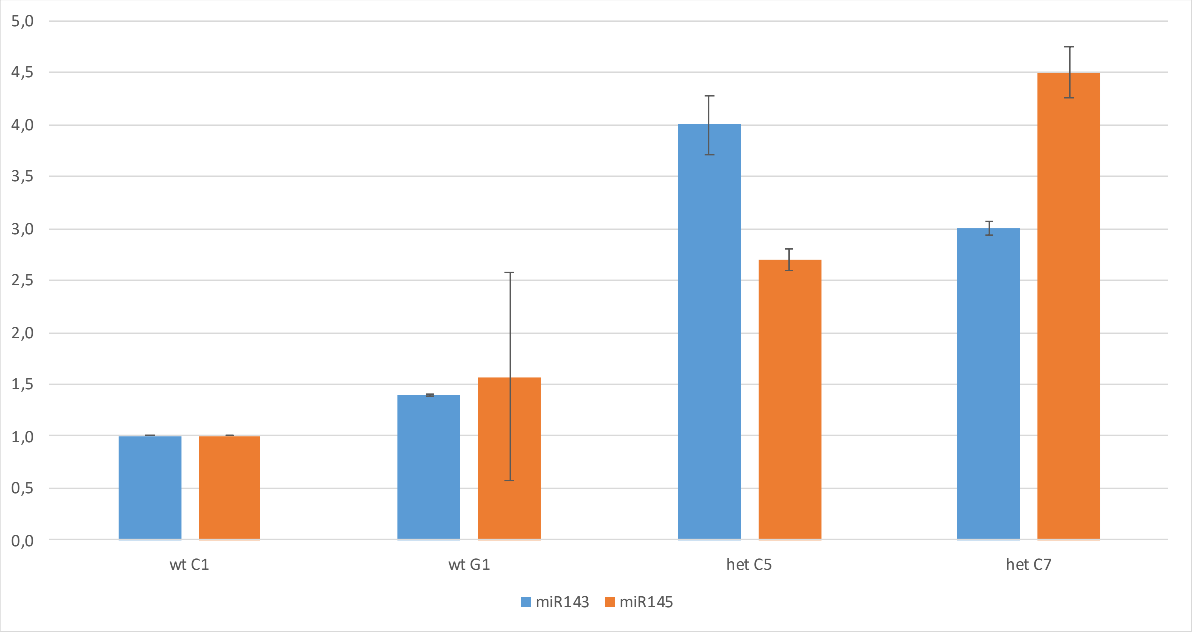

Figure 1 for Referee1. RT-qPCR analysis for miR-143 and -145 in wt and het clones.

5. Figure 5: the two different bars in panel B refer to different clones or technical replicates?

We apologize if the figure was not clear. In Figure 5B (now 6B), we report the level of expression of miR-143 and -145 in the same two clones (G- and A-allele) at different culture passages, with the scope of evaluating whether the difference of miRNA expression was dependent on a particular moment of the cell passage or, as then resulted, a specific characteristic due to the presence of the variation. In order to enhance the clarity of the message, we have now modified the figure.

6. A major strength of the study involves the two separate and robust clinical cohorts (>1000 pts). Within these cohorts, significant differences in prevalence did exist (e.g. Neapolis: diabetes, CKD) between SNP carriers that are not easily explained by a smooth muscle phenotype. These should be amply discussed.

We thank the referee for the observation, and we have now better explained and discussed these results (page13, line 4; page 15, lines 14-15). We agreed that the two cohorts show some clinical differences, and from the statistical point of view diabetes is a major one (G-allele vs A-allele: $p<0.01$). However, the role of rs41291957 and diabetes was not further analyzed because, from an etiological point of view, this pathology is not related to smooth muscle cell biology. For instance, the variation might play a role in the biology of tissues related to insulin production or resistance, but this is out-of-scope for the present work. Indeed, in the Neapolis cohort, the difference is nominally significant, but it does not survive multiple-testing correction. For both cohorts, we ran models that considered diabetes as covariate. Therefore, according to the specific anatomical assessment of CAD phenotype, in the Neapolis study the overall cohort of patients was CTO-stratified, as reported in Figure 8 and Table EV4. Of note, this multivariate analysis demonstrated that this association (rs41291957-CTO) was independent, as explained above, from other potential confounders, including diabetes. Furthermore, the difference is not significant in LURIC at all.

7. The authors should discuss more extensively their own previous findings in miR-143/145 knockout/overexpressing mice as to avoid inclusion of an animal model in the present manuscript.

We thank the referee for the suggestion, but we did not include any animal studies because, in our opinion, they are out of scope for the present study. Indeed, since the genetic variation is related to the human genome, the use of an animal model would not be informative. Also, introduce the variation into the mouse genome require further years of work. Similarly, the use of a knockout animal would not be informative. These are the main reasons that prompted us to not study rs41291957 in animal models. We have discussed the point raised by the reviewer (pag 16, lines 2-7).

Minor

1. Consider rephrasing second sentence of Abstract: "... has been established not only for gene products but also for microRNAs (miRNAs)". Gene products probably refers to protein coding genes and microRNAs refers to microRNA genes or genes encoding microRNAs.

We thank the referee for this suggestion, and have modified the text as requested: “predisposition has been established not only for protein coding genes but also for genes encoding microRNAs (miRNAs).”

2. Rephrase sentence on page 14: "Secondly, the retrospective nature the clinical studies..."

We thank the referee for the suggestion, and have rephrased the sentence as: “the retrospective analysis of the clinical studies…”

Referee #2

Comments on Novelty/Model System for Author

the paper is technically very nicely executed but to me it lacks novelty (just another snp regulating a miRNA) and medical impact (there is no "medicine" in the paper)

Remarks for Author

In this manuscript, Hall and colleagues add new interesting insights to the role of single nucleotide polymorphisms (SNPs) to coronary artery disease. The authors focus here on rs41291957 which upregulates miR-143 and miR-145, triggering the phenotypic switch of vascular smooth cells towards a more contractile phenotype. Furthermore, the authors propose a novel link between the expression of rs41291957 and a protective status in human CAD. Although other SNPs as rs353292 and rs4705343 have been shown to modulate the miRNA cluster miR143/145, this work in itself is novel. While the manuscript of Hall et al. is carefully executed and technically sound, however I recommend the authors to address the following points.

We thank the referee for his/her positive comments, but it is not clear to us the different tone between the statements of “Comments on Novelty/Model System for Author” and “Remarks for Author”.

Minor comments

1. Figure 3B: The authors should try improving the quality of the pictures. Maybe with a higher magnification. Also, DAPI should be included as it is done in other figures for quantification.

We apologize for the low quality of the pictures (probably there was a problem with the PDF conversion) and for not having included DAPI staining. We have now modified the images (New Figure 4B) and included all requested information (Appendix Figure S2A).

2. Figure 4C: The authors should include DAPI staining pictures and bar scale information.

The specific images are included in Appendix Figure S2B.

3. Figure 5: The effects in SMC differentiation are only moderate. The authors may consider including further differentiation markers or longer time points to enhance the effects.

We thank the referee for the comment. However, in terms of phenotypic properties, such as proliferation, cell size, actin organization and contraction capacity, we think that the observed effects are consistent with a greater differentiation status. We must consider, as reported by several groups including ours, that the level of both miRNAs in smooth muscle cells is very high; thus, an increase such as the one observed might be very close to a saturation point for the modulation of the cellular phenotype. Therefore, it is most unlikely to trigger a further differentiation effect on these cells. As a matter of fact, we tried to measure these features also at further cellular passages, but we did not observe any major difference compared to the data presented.

Of note, as requested by the referee, we have now added further differentiation markers in the protein analysis (Figure 6G and EV3B).

4. Figure 5 H: The Western blots should be n=3 and quantified. The authors should consider including further contractile markers (e.g. calponin, caldesmon, sm-actin).

As said above, we have now included other contractile markers and the quantification (Figure 6G and EV3B).

5. Figure 6.F. the Western blot would need to be n=3 and be quantified for normalization to GAPDH.

We apologize for not having included the quantification, which we have now given in Figure EV4B. .

6**. Please correct "Collagene" for collagen: Fig5L and Fig6H**

We have modified the text.

Referee #3

Comments on Novelty/Model System for Author

The findings are novel and clearly of interest to the field. The statistical analysis might benefit from an additional statistical review.

We thank the referee for the positive comment. We have now included an extended paragraph for statistical analysis related to the in vitro experiments (pag. 18 and 19).

Remarks for Author

In this work Hall and colleagues elegantly analysed the effect of a single nucleotide polymorphism (SNP) identified in the miR-143/145 cluster gene. Date reported in their work indicated that the SNP with a minor allele frequency of at least 10% was protective in coronary artery disease (CAD) patients. The miR-143/145 cluster is predominantly expressed in vascular smooth muscle cells and regulates their phenotypic switch, crucial in several cardiovascular diseases, such as atherosclerosis, CAD, and arterial hypertension.
The work is very well presented, and experiments have been properly designed and discussed. The rationale is clear, and data here reported support the existence of a SNP able to confer protection in certain patients affected by artery disease. This reviewer has some concerns that authors should address to improve their work:

1. Why did the authors, among all miRNAs and miRNA clusters that are known to play key roles in CAD decide for the miR-143/145 cluster? This point should be better outlined and discussed in the introduction. Here, the authors should include other relevant miRNAs, for which SNP have been identified to underline the rationale behind the selection of miR-143/145 cluster. Indeed, athero-prone miRNAs are known to contain SNPs and not all have been studied so far.

We thank the referee for his/her point of discussion. Actually, this cluster is probably the most relevant in controlling SMC phenotypic switch in vascular pathologies, as demonstrated by several groups, including ours. In the past, we concentrated our attention on the epigenetic regulation of these two miRNAs, also generating genetically modified animal models. Here, we decided to investigate whether human genetic variations might impact its regulation. However, we agree with the reviewer on the need to better discuss our choice in the introduction (pag. 4, lines 15-16; pag. 5, lines 1-5).

2. The authors identified the rs41291957 SNP. The ID correspond to two different mutations reported, namely 148808390 G>A and 149428927 G>A. Which one are authors are referring to? What is the frequency and difference (if any) between the two?

Probably, the referee is referring to data reported in the NCBI database (https://www.ncbi.nlm.nih.gov/snp/rs41291957). Actually, there are two genomic location entries identifying the same variation in that database: specifically, 148808390 refers to human genome version GRCh37.p13, while 149428927 refers to human genome version GRCh38.p12. Thus, practically there is no difference in the two cited codes, and every presence frequency of the variation must be refereed only to the general ID rs41291957, data already included in the paper.

3. Authors indicated that the G>A mutation generates a potential less complex secondary structure of the miRNA precursor, leading to an increased accessibility for the miRNA maturation enzymes. However, the prediction obtained is merely computational and based on a centromeric prediction. Authors should explain the parameters used in more detail. First, did authors considered the G:U pairs? What is the rationale of a centroid prediction instead of a minimum free energy secondary structure?

As indicated by the referee, we predicted secondary structures, hypothesizing different structural organization for the WT and SNP pri-miRNAs. The reason for using centroid prediction is merely based on the fact that this is one of the most accurate tools for predicting RNA secondary structures (10.1016/j.ymeth.2016.04.004; 10.3389/fgene.2011.00054; 10.1093/bioinformatics/btn60). While most algorithms that computationally predict RNA folding patterns are based on minimizing free energy, many do not account for protein interactions, evolutionary sequence conservation, or RNA dynamics. In addition, the fidelity of *in silico* prediction is known to decrease as RNA sequence length increases (>400nt). Thus, a more reliable method is highly preferred, such as the centroid method. The centroid prediction in based on Boltzmann-weighted structure ensemble and, in comparison with the minimum free energy (MFE) structures, its outputs make 30% fewer prediction errors, as measured by the positive predictive value (PPV), while a marginal improvement on sensitivity has been calculated (10.1261/rna.2500605).
As the referee can observe on the enclosed image (Figure 1 for Referee3), we of course considered G:U pairs (the structures in Figure 1B and 1C show also G:U pairs). As for the parameters used, we utilized the default ones indicated by the RNAfold algorithm. We have now included this information in the EV method section (pag 36, lines 7-11).

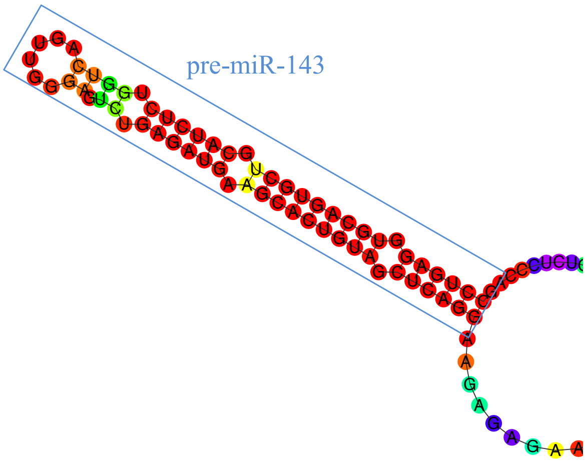

Figure 1 for Referee3. Magnification of image of figure 1B, showing G:U pairs.

4. On a related note and to support the hypothesis of a different accessibility of the enzyme at the secondary structure, the authors should perform an enzymatic cleavage of single strand RNA, followed by a sequencing or similar of the RNA:RNA secondary structures. The loops should be protected from the cleavage and show the differences in the secondary structures. Ideally, the authors should perform an immunoprecipitation to see if any difference occurs in the binding affinity in G>A group. This is fundamental to support the RNA secondary structure accessibility and prediction.

We thank the referee for this very important comment. We indeed performed a cleavage assay (Figure 2A) to evaluate whether a real difference in secondary structure exists between the G- and A-allele pri-miR-143/145. In order to do so, we transcribed both primary miRNAs in vitro and treated them with RNAse I, an enzyme that digests single strand RNA; the loops should be more subject to degradation. RNAs were subjected first to tapestation analysis (an automatized system to run fragments on an acrylamide gel capillary), which already revealed a difference in size between the digested G- and A-allele pri-miRNAs (Figure EV2), confirming the putative role of rs41291957 in generating a more accessible structure. The two RNAs were then subjected to RNA sequencing, which confirmed the difference in secondary structures, as observed by the % of enriched digested reads along the G- or A-allele sequence (Figure 2B). Furthermore, the lengths of the obtained reads were shorter for the A-allele (Figure 2C), corroborating the increased digestion rate observed in the tapestation analysis. Finally, to obtain statistical data, we performed further cleavage experiments (n=6) and then run qPCR analysis for the pri-miR-143/145, confirming the difference in terms of degradation between the G- and A-allele (Figure 2D). Moreover, we have tried several times to perform IP for Drosha in HEK293 cells transiently expressing the G- or A-allele pri-miR-143/145, but, unfortunately, we were not able to efficiently precipitate the complex.
Nonetheless, we believe that the new provided data about the altered secondary structure strongly support our hypothesis.

5. Statistical analysis should be included and better elaborated in the material and methods as paragraph, and not only in the figure legends.

We apologize if the statistical analysis was not clearly described. We have now included an extended paragraph on statistical analysis related to the in vitro experiment (pag. 18 and 19).

6. The data reported in Figure 4B indicated that C10 clone significantly reduced miR-145 but not miR-143 levels. How do author explain this?

We thank the referee for the observation; however, this is very difficult to answer. As these are genetically modified cells, and although the CRISPR system that we employed utilized two different gRNA guides in order to reduce the possibility of random mutation, we cannot exclude this occurrence. Therefore, the difference might depend upon the modulation of other particular regulatory mechanisms, which we cannot anticipate. Nonetheless, the referee must consider that, ultimately, both WT clones had miR-145 expression levels that were lower compared to the SNP ones, strongly suggestive of the bona fide of our conclusion. The statistical analysis was performed considering all raw data derived from the qPCR experiments; thus, for the DDct calculation we selected as 1 only one sample (referred to as the C1 clone), and this generated the difference seen between the two WT clones. Overall, we can only speculate on the presence of reduced expression of miR-145 in the C10 clone. The important message here is that, independently from this difference, the SNP clones had always higher levels of expression of both miRNAs compared to the controls. On the other hand, just for the referee's evaluation, we are including expression data of one more WT clone selected during the CRISPR screening (Figure 2 for Referee 3). The C1 and C10 clones were chosen because they had phenotypic characteristics similar to the original cells. As the referee can observe, the WT clone G1, despite some variability, has even a small increase of miR-145 compared to C1.

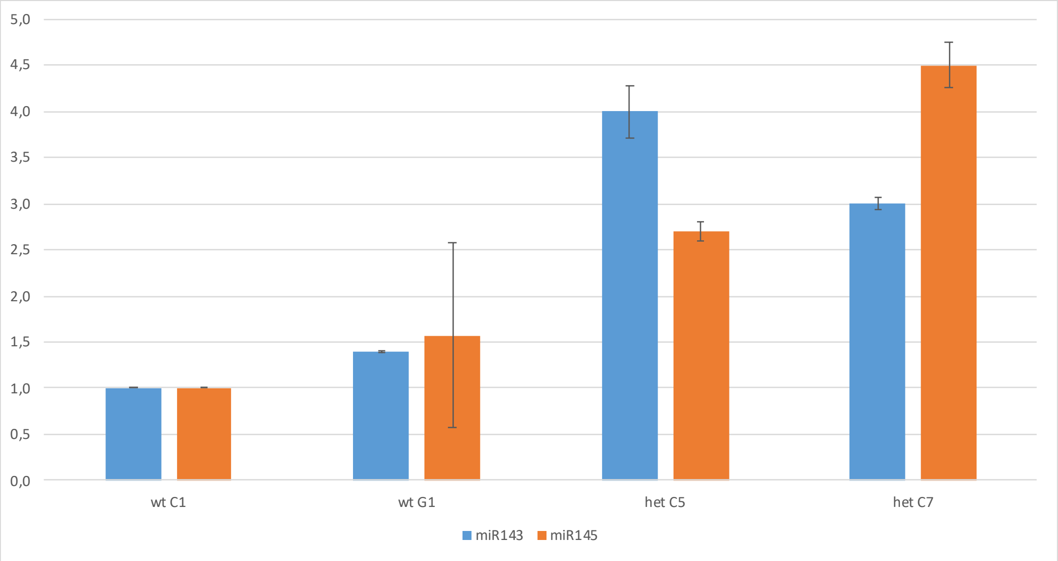

Figure 2 for Referee 3. RT-qPCR analysis for miR-143 and -145 in wt and het clones.

7. The miR-143 and miR-145 can play different roles. Which is the effect of the SNP on their individual function?

We thank the referee for the comment. Initially, we did not run these experiments, because we believed that the combined effect of both miRNAs is important to the biology of VSMCs carrying the A-allele. In order to demonstrate this hypothesis, we have now, as requested by the referee, run rescue experiments, inhibiting miR-143 or -145 separately. We did not observe complete recovery of all biological features, as measured in A-allele VSMCs treated with both i143 and i145. The single miRNA inhibition rescued proliferation and stress fiber formation, but not the increased cell size (Figure EV5). Overall, the new set of experiments further supports our hypothesis of the miR-SNP acting on miR-143/145 rather than on one of the two miRNAs separately.

8. The authors focused on GA in heterozygosis. However, AA homozygosis have been reported in patients. The authors should explain the reason for focusing on heterozygosis and introduce the effect of AA homozygosis. This could be particularly important with regards to future potential therapies aiming the analysis of the SNP in other diseases and cell types. Related to this, the authors describe the AA + GA data from patients but they did not investigate it in vitro. These data would be very instructive concerning the existence of patients carrying the AA homozygosis. Moreover, how do authors explain the higher rate of diabetes? This should be discussed or ideally studied, at least in vitro.

We thank once more the referee for the useful comment. We would like to underline that in a study population analysis, the combined hetero/homozygous data are considered. In our specific case, the percentage of AA patients is limited in the two populations: specifically, in the Neapolis study there were 17 out of 327 (5.19%), while in LURIC only 19 patients carried the AA allele out of 532 (3.5%). It is clear that with such frequency no statistical conclusion can be drawn. On the other hand, for the in vitro study our aim was to obtain homozygous and heterozygous cellular systems: however, we successfully obtained only cells with the latter genotype. Specifically, the CRISPR/Cas9 approach required several months of setting up and experimental work. During this period, we screened and sequenced a substantial number of clones, identifying only few heterozygous ones, which were then employed for the study. These difficulties were even amplified when we tried to apply such an approach to primary

human VSMCs. For this reason, we took advantage of a previous analysis made by Prof. Quertermous' laboratory (Stanford University), who very kindly shared with us their data, and among their sequenced lines we identified two generated by the same vendor, one of which with the G/A polymorphism. The genotypes were then further validated by our lab. This part of the work also required a very long time to be concluded. Thus, for all these reasons, we were not able to test the homozygous variation in vitro. Nonetheless, considering also the very low prevalence of AA patients, we believe that this kind of experiment would not add any further clinical value to our findings.
Regarding the higher rate of diabetes in the Neapolis cohort, we do not consider this clinical feature important for the present study, which focuses on the biology of vascular smooth muscle cells. Specifically, the two cohorts show some clinical differences, and from the statistical point of view, diabetes is a major one (G-allele vs A-allele: $p<0.01$). In the Neapolis cohort, the difference is nominally significant, but it does not survive multiple-testing correction. For both cohorts, we ran models considering diabetes as covariate. Indeed, according to the specific anatomical assessment of CAD phenotype, in the Neapolis study the overall cohort of patients was CTO-stratified, as reported in Figure 8 and Table EV4. Of note, this multivariate analysis demonstrated that this association (rs41291957-CTO) was independent from other potential confounders, including diabetes. Furthermore, the difference is not significant in LURIC at all. In general, the role of rs41291957 and diabetes was not further analyzed because, from an etiological point of view, this pathology is not related to smooth muscle cell biology. For instance, the variation might play a role in the biology of tissues related to insulin production or resistance, but this is out-of-scope for the present work. We have now better explained and discussed these results (pag. 13, line 4; pag 15, line 14-15).

9. There is a difference between C5 and C7 clones on miR-143 and miR-145 expression, which is opposite. How can authors explain it? How can they have the final effect on GFP expression? is there any target saturation? Do the miRNAs show a competitive role in terms of co-factor accessibility?

We thank the referee for the observation. As he/she might note, although different, the level of both miRNAs is always higher compared to the WT clones; this is, in our opinion, the fundamental message. The reason for this is actually difficult to understand, but again, we must consider that we have worked with genetically modified cells, in which there might be some unexpected effects due to genomic correction.
Regarding GFP expression, we apologize if the experimental setting was not properly described. Actually, we employed the same system that was described in Figure 4A, in which two tandem and complimentary sequences for miR-143 or -145 were cloned as synthetic 3'UTRs of the GFP gene. Thus, when the miRNA binds its target there is a decay of the target itself, in this case the GFP. Of course, the level of target inhibition depends on the amount of produced miRNA. Then, in this particular case, these constructs (decoy-miR-143 and decoy-miR-145) were transfected in the CRISPR clones and GFP level measured (normalized by the number of cells). Specifically, the two employed systems were independent and there was no competition of the two miRNAs for the target, because we used decoy-143 and -145 separately. Furthermore, there was no competition between the two miRNAs for co-factor accessibility, because the RISC complex works based on complementarity between the target mRNA – in this case GFP-3'UTR/Decoy-miR-143 or GFP-3'UTR/Decoy-miR-145 – and the miRNAs themselves, so there is a very high specificity for the two systems.
We have now better described the experimental results (Pag 10, lines 11-17).

10. What is the final conclusion that the authors would draw from the existence of the SNP? Is the frequency of the SNP the same in all different populations studied?

The final conclusion from our study is that the A-allelic variant promotes a more differentiated phenotype in human VSMCs by directly modulating the expression of miR-143 and miR-145. Collectively, these findings might explain the protective role of the A-allele in the reduction of CAD events in two large independent patient cohorts.
The SNP frequency in both populations is very similar: 18.94% in Neapolis and 20.4% in LURIC.

Minor concern:
1. Page 6, line 3: ensemble should be changed with ensembl
We have fixed the text.

2. The legend of figure 6A is missing.
We have fixed the legend.

1st Revision - Editorial Decision 18th Aug 2021

18th Aug 2021

Dear Prof. Elia,

Thank you for the submission of your revised manuscript to EMBO Molecular Medicine. I am pleased to inform you that we will be able to accept your manuscript pending the following final amendments:

1) In the main manuscript file, please do the following:
- Correct/answer the track changes suggested by our data editors by working from the attached document.
- Add callouts for Figure 2C and Figure 7F.
- In M&M, include a statement that in addition to the principles set out in the WMA Declaration of Helsinki the experiments also conformed the Department of Health and Human Services Belmont Report.
- In addition to the accession number please provide URL for deposited datasets. Please be aware that all datasets should be made freely available upon acceptance, without restriction. Use the following format to report the accession number of your data:

The datasets produced in this study are available in the following databases:
[data type]: [full name of the resource] [accession number/identifier] ([doi or URL or identifiers.org/DATABASE:ACCESSION])

Please check "Author Guidelines" for more information.
https://www.embopress.org/page/journal/17574684/authorguide#availabilityofpublishedmaterial
2) Expanded View file: Please move M&M to the main manuscript (including references) and upload tables as separate files.
3) Dataset: Please add title/legend in a separate tab.
4) Source data: Please upload one file per figure.
5) Synopsis: Please check your synopsis text and image, revise them if necessary and submit their final versions with your revised manuscript. Please be aware that in the proof stage minor corrections only are allowed (e.g., typos).
- Synopsis image: Please resize the synopsis image to 550 px-wide x (250-400)-px high and submit it as a high-resolution jpeg file.
6) For more information: There is space at the end of each article to list relevant web links for further consultation by our readers. Could you identify some relevant ones and provide such information as well? Some examples are patient associations, relevant databases, OMIM/proteins/genes links, author's websites, etc...
7) As part of the EMBO Publications transparent editorial process initiative (see our Editorial at http://embomolmed.embopress.org/content/2/9/329), EMBO Molecular Medicine will publish online a Review Process File (RPF) to accompany accepted manuscripts. This file will be published in conjunction with your paper and will include the anonymous referee reports, your point-by-point response and all pertinent correspondence relating to the manuscript. Let us know whether you agree with the publication of the RPF and as here, if you want to remove or not any figures from it prior to publication. Please note that the Authors checklist will be published at the end of the RPF.
8) Please provide a point-by-point letter INCLUDING my comments as well as the reviewer's reports and your detailed responses (as Word file).

I look forward to reading a new revised version of your manuscript as soon as possible.

Yours sincerely,

Zeljko Durdevic

Zeljko Durdevic
Editor
EMBO Molecular Medicine

***** Reviewer's comments *****

Referee #1 (Comments on Novelty/Model System for Author):

The relevant models were used, the authors now also discussed and justified the absence of an animal model

Referee #1 (Remarks for Author):

no further questions

Referee #3 (Remarks for Author):

The manuscript has been suitably revised and is now acceptable for publication.

The authors performed the requested changes.

2nd Revision - Editorial Decision 24th Aug 2021

We are pleased to inform you that your manuscript is accepted for publication and is now being sent to our publisher to be included in the next available issue of EMBO Molecular Medicine.

EMBO PRESS

YOU MUST COMPLETE ALL CELLS WITH A PINK BACKGROUND

PLEASE NOTE THAT THIS CHECKLIST WILL BE PUBLISHED ALONGSIDE YOUR PAPER

Corresponding Author Name: Leonardo Elia
Journal Submitted to: EMBO Molecular Medicine
Manuscript Number: EMM-2021-14060

Reporting Checklist For Life Sciences Articles (Rev. June 2017)

This checklist is used to ensure good reporting standards and to improve the reproducibility of published results. These guidelines are consistent with the Principles and Guidelines for Reporting Preclinical Research issued by the NIH in 2014. Please follow the journal's authorship guidelines in preparing your manuscript.

A- Figures
1. Data
The data shown in figures should satisfy the following conditions:

- the data were obtained and processed according to the field's best practice and are presented to reflect the results of the experiments in an accurate and unbiased manner.
- figure panels include only data points, measurements or observations that can be compared to each other in a scientifically meaningful way.
- graphs include clearly labeled error bars for independent experiments and sample sizes. Unless justified, error bars should not be shown for technical replicates.
- if n< 5, the individual data points from each experiment should be plotted and any statistical test employed should be justified
- Source Data should be included to report the data underlying graphs. Please follow the guidelines set out in the author ship guidelines on Data Presentation.

2. Captions

Each figure caption should contain the following information, for each panel where they are relevant:

- a specification of the experimental system investigated (eg cell line, species name).
- the assay(s) and method(s) used to carry out the reported observations and measurements
- an explicit mention of the biological and chemical entity(ies) that are being measured.
- an explicit mention of the biological and chemical entity(ies) that are altered/varied/perturbed in a controlled manner.

- the exact sample size (n) for each experimental group/condition, given as a number, not a range;
- a description of the sample collection allowing the reader to understand whether the samples represent technical or biological replicates (including how many animals, litters, cultures, etc.).
- a statement of how many times the experiment shown was independently replicated in the laboratory.
- definitions of statistical methods and measures:
  - common tests, such as t-test (please specify whether paired vs. unpaired), simple $\chi 2$ tests, Wilcoxon and Mann-Whitney tests, can be unambiguously identified by name only, but more complex techniques should be described in the methods section;
  - are tests one-sided or two-sided?
  - are there adjustments for multiple comparisons?
  - exact statistical test results, e.g., P values = x but not P values < x;
  - definition of 'center values' as median or average;
  - definition of error bars as s.d. or s.e.m.

Any descriptions too long for the figure legend should be included in the methods section and/or with the source data.

In the pink boxes below, please ensure that the answers to the following questions are reported in the manuscript itself. Every question should be answered. If the question is not relevant to your research, please write NA (non applicable). We encourage you to include a specific subsection in the methods section for statistics, reagents, animal models and human subjects.

USEFUL LINKS FOR COMPLETING THIS FORM

http://www.antibodypedia.com
http://1degreebio.org
http://www.equator-network.org/reporting-guidelines/improving-bioscience-research-repor
http://grants.nih.gov/grants/olaw/olaw.htm
http://www.mrc.ac.uk/Ourresearch/Ethicsresearchguidance/Useofanimals/index.htm
http://ClinicalTrials.gov
http://www.consort-statement.org
http://www.consort-statement.org/checklists/view/32-consort/66-title
http://www.equator-network.org/reporting-guidelines/reporting-recommendations-for-tumo
http://datadryad.org
http://figshare.com
http://www.ncbi.nlm.nih.gov/gap
http://www.ebi.ac.uk/ega
http://biomodels.net/
http://biomodels.net/miriam/
http://jjj.biochem.sun.ac.za
https://osp.od.nih.gov/biosafety-biosecurity-and-emerging-biotechnology/
http://www.selectagents.gov/

B- Statistics and general methods

Please fill out these boxes (Do not worry if you cannot see all your text once you press return)

1.a. How was the sample size chosen to ensure adequate power to detect a pre-specified effect size?	N/A
1.b. For animal studies, include a statement about sample size estimate even if no statistical methods were used.	N/A
2. Describe inclusion/exclusion criteria if samples or animals were excluded from the analysis. Were the criteria pre-established?	N/A
3. Were any steps taken to minimize the effects of subjective bias when allocating animals/samples to treatment (e.g. randomization procedure)? If yes, please describe.	N/A
For animal studies, include a statement about randomization even if no randomization was used.	N/A
4.a. Were any steps taken to minimize the effects of subjective bias during group allocation or/and when assessing results (e.g. blinding of the investigator)? If yes please describe.	NO
4.b. For animal studies, include a statement about blinding even if no blinding was done	N/A
5. For every figure, are statistical tests justified as appropriate?	YES
Do the data meet the assumptions of the tests (e.g., normal distribution)? Describe any methods used to assess it.	F-test
Is there an estimate of variation within each group of data?	NO

Is the variance similar between the groups that are being statistically compared?	YES

C- Reagents

6. To show that antibodies were profiled for use in the system under study (assay and species), provide a citation, catalog number and/or clone number, supplementary information or reference to an antibody validation profile. e.g., Antibodypedia (see link list at top right), 1DegreeBio (see link list at top right).	We provided all catalog number in the m&m section
7. Identify the source of cell lines and report if they were recently authenticated (e.g., by STR profiling) and tested for mycoplasma contamination.	We provided all information, including catalog number in the m&m section

* for all hyperlinks, please see the table at the top right of the document

D- Animal Models

8. Report species, strain, gender, age of animals and genetic modification status where applicable. Please detail housing and husbandry conditions and the source of animals.	N/A
9. For experiments involving live vertebrates, include a statement of compliance with ethical regulations and identify the committee(s) approving the experiments.	N/A
10. We recommend consulting the ARRIVE guidelines (see link list at top right) (PLoS Biol. 8(6), e1000412, 2010) to ensure that other relevant aspects of animal studies are adequately reported. See author guidelines, under 'Reporting Guidelines'. See also: NIH (see link list at top right) and MRC (see link list at top right) recommendations. Please confirm compliance.	N/A

E- Human Subjects

11. Identify the committee(s) approving the study protocol.	N/A
12. Include a statement confirming that informed consent was obtained from all subjects and that the experiments conformed to the principles set out in the WMA Declaration of Helsinki and the Department of Health and Human Services Belmont Report.	We included such statment in paragraph dedicated to the description of the patient populations
13. For publication of patient photos, include a statement confirming that consent to publish was obtained.	N/A
14. Report any restrictions on the availability (and/or on the use) of human data or samples.	N/A
15. Report the clinical trial registration number (at ClinicalTrials.gov or equivalent), where applicable.	N/A
16. For phase II and III randomized controlled trials, please refer to the CONSORT flow diagram (see link list at top right) and submit the CONSORT checklist (see link list at top right) with your submission. See author guidelines, under 'Reporting Guidelines'. Please confirm you have submitted this list.	N/A
17. For tumor marker prognostic studies, we recommend that you follow the REMARK reporting guidelines (see link list at top right). See author guidelines, under 'Reporting Guidelines'. Please confirm you have followed these guidelines.	N/A

F- Data Accessibility

18: Provide a "Data Availability" section at the end of the Materials & Methods, listing the accession codes for data generated in this study and deposited in a public database (e.g. RNA-Seq data: Gene Expression Omnibus GSE39462, Proteomics data: PRIDE PXD000208 etc.) Please refer to our author guidelines for 'Data Deposition'.<br><br>Data deposition in a public repository is mandatory for:<br>a. Protein, DNA and RNA sequences<br>b. Macromolecular structures<br>c. Crystallographic data for small molecules<br>d. Functional genomics data<br>e. Proteomics and molecular interactions	We included the accesion code of our sequencing data
19. Deposition is strongly recommended for any datasets that are central and integral to the study; please consider the journal's data policy. If no structured public repository exists for a given data type, we encourage the provision of datasets in the manuscript as a Supplementary Document (see author guidelines under 'Expanded View' or in unstructured repositories such as Dryad (see link list at top right) or Figshare (see link list at top right).	N/A
20. Access to human clinical and genomic datasets should be provided with as few restrictions as possible while respecting ethical obligations to the patients and relevant medical and legal issues. If practically possible and compatible with the individual consent agreement used in the study, such data should be deposited in one of the major public access-controlled repositories such as dbGAP (see link list at top right) or EGA (see link list at top right).	N/A
21. Computational models that are central and integral to a study should be shared without restrictions and provided in a machine-readable form. The relevant accession numbers or links should be provided. When possible, standardized format (SBML, CellML) should be used instead of scripts (e.g. MATLAB). Authors are strongly encouraged to follow the MIRIAM guidelines (see link list at top right) and deposit their model in a public database such as Biomodels (see link list at top right) or JWS Online (see link list at top right). If computer source code is provided with the paper, it should be deposited in a public repository or included in supplementary information.	N/A

G- Dual use research of concern

22. Could your study fall under dual use research restrictions? Please check biosecurity documents (see link list at top right) and list of select agents and toxins (APHIS/CDC) (see link list at top right). According to our biosecurity guidelines, provide a statement only if it could.	N/A
